# Trends in High-Risk Sexual Behaviors among General Population Groups in China: A Systematic Review

**DOI:** 10.1371/journal.pone.0079320

**Published:** 2013-11-13

**Authors:** Rui Cai, Jan Hendrik Richardus, Caspar W. N. Looman, Sake J. de Vlas

**Affiliations:** 1 Department of Public Health, Erasmus MC, University Medical Center, Rotterdam, The Netherlands; 2 Student of China Scholarship Council, Beijing, China; University of Texas Health Science Center San Antonio Texas, United States of America

## Abstract

**Background:**

The objective of this review was to investigate whether Chinese population groups that do not belong to classical high risk groups show an increasing trend of engaging in high-risk sexual behaviors.

**Methods:**

We systematically searched the English and Chinese literature on sexual risk behaviors published between January 1980 and March 2012 in PubMed and the China National Knowledge Infrastructure (CNKI). We included observational studies that focused on population groups other than commercial sex workers (CSWs) and their clients, and men who have sex with men (MSM) and quantitatively reported one of the following indicators of recent high-risk sexual behavior: premarital sex, commercial sex, multiple sex partners, condom use or sexually transmitted infections (STIs). We used generalized linear mixed model to examine the time trend in engaging in high-risk sexual behaviors.

**Results:**

We included 174 observational studies involving 932,931 participants: 55 studies reported on floating populations, 73 on college students and 46 on other groups (i.e. out-of-school youth, rural residents, and subjects from gynecological or obstetric clinics and premarital check-up centers). From the generalized linear mixed model, no significant trends in engaging in high-risk sexual behaviors were identified in the three population groups.

**Discussion:**

Sexual risk behaviors among certain general population groups have not increased substantially. These groups are therefore unlikely to incite a STI/HIV epidemic among the general Chinese population. Because the studied population groups are not necessarily representative of the general population, the outcomes found may not reflect those of the general population.

## Introduction

Sexually transmitted infections (STIs) have been recognized as a major public health problem in China since they re-emerged with the introduction of the ‘Open-Door Policy’ in 1979 [1]. STIs such as syphilis and gonorrhea were considered to have been eliminated in the 1960s by massive screening and free treatment. However, they are now among the most commonly reported notifiable diseases [2]. The rate of new syphilis cases increased from 0.09 in 1990 to 5.08 in 2000, and on to 26.86 per 100,000 population in 2010 [3]. Although the trend for gonorrhea showed an increasing and declining pattern, the incidence of gonorrhea remains relatively high, with over 90,000 new cases reported in 2012 [3]. The number of reported new HIV cases stayed relatively low, with almost no cases between 1990 and 2000, and subsequently an increase to 1.20 cases per 100,000 population in 2010 [3]. In general, the epidemic of STIs/HIV in China has expanded over the past decades.

The resurgence of STIs can largely be explained by the demographic and social changes in China since the introduction of the ‘Open-Door Policy’. Commercial sex workers (CSWs) and their clients, and men who have sex with men (MSM) are considered as the classical high risk groups of STIs. The size of these high risk groups has increased considerably in China over the past three decades. For instance, the number of CSWs is currently estimated to be between one and four million [4]–[7]. The flourishing commercial sex industry has provided a channel for the spread of STIs. There have also been substantial changes for population groups not belonging to the classical high risk groups. Firstly, the restriction on population movement was relaxed. Many people now migrate across the country in search of work, particularly from rural to urban areas. The number of this so-called ‘floating population’ that were not registered in the cities to which they have migrated, was estimated to be over 200 million in 2012 [8]. The majority of this so-called ‘floating population’ comprises young male adults, who are sexually active and have limited education, relatively low social status, and limited access to health services [9]. Due to these conditions and the separation from family members, a larger proportion of the floating population may participate in high-risk sexual behaviors, such as commercial sex, compared to the non-migrant population. Secondly, Western culture has penetrated the society, and may have exerted an influence on people’s attitudes towards sexuality. It is believed that in particular the young generation such as college students has become more tolerant of commercial sex, premarital sex, casual sex, and multiple sexual partners [2], [10].

Given the social developments presented above, it may be assumed that an increasing proportion of the Chinese population has opted to engage in high-risk sexual behaviors that might facilitate the spread of STIs, including HIV. Studies on changes in the proportion of people involved in high-risk sexual behaviors in China have predominately focused on CSWs and their clients, STI clinic attendees, men who have sex with men, former paid blood donors, and injecting drug users. Indeed, some groups showed increasing engagement in high-risk sexual behaviors and a corresponding increase in the STIs/HIV prevalence [5], [11]–[13]. To date, however, reviews on sexual risk behaviors among other, more general population groups are hardly available. One review provided data about sexual practices among the general population [14], but it only included data up to 2003 and without any description of possible trends. It is therefore unclear if general population groups engage increasingly in sexual risk behaviors.

The general population cannot be considered the key driver of the STIs/HIV epidemic in China as is the case with the classical high-risk groups, but transmission of STIs/HIV will be very difficult to stop if high-risk sexual behaviors become common among the general population. To direct STIs/HIV prevention efforts, knowledge on trends in engaging in high-risk sexual behaviors, including not using condoms, among the general population is needed. The aim of this systematic review is to investigate possible trends of engaging in high-risk sexual behaviors (i.e. premarital sex, multiple sex partners, and commercial sex) by general population groups, in particular the floating population and college students. The review is structured by category of population groups and indicator of high-risk sexual behaviors.

## Methods

### Search Strategy

We conducted a comprehensive literature search in both PubMed and its Chinese equivalent, the Chinese National Knowledge Infrastructure (CNKI). The latter is the most comprehensive database in Chinese scientific literature; it includes studies published in a wide range of periodicals, conference proceedings, and newspapers. Based on a bibliometric method, China has a core journal selection system that creates a sub-database of journals that have the latest professional information and been formally peer-reviewed [15]. Given the large number of eligible studies, we narrowed our search to the core journals selected within the CNKI, because the sub-database was likely to identify the majority of relevant studies. We retrieved studies published from January, 1980 to March, 2012, in both Chinese and English languages. The search terms included both Mesh terms: ‘sexual behavior’ and ‘sexually transmitted diseases’, and full text words: ‘condoms, ‘commercial sex’, ‘multiple sex partner’, ‘sexually transmitted infections’ and ‘China’. We used the term ‘sexually transmitted infections’, because the occurrence of STIs can be considered an indicator to describe consequence of relatively recent high-risk sexual behaviors. The detailed search string can be found in File S1.The search terms did not have any restriction about the population groups, as it is hard to find a suitable keyword for the study population groups that we target; those who do not belong to the classical high risk groups. We initially included all population groups in our literature search and excluded the groups that are at highest risk of STIs/HIV manually in the study selection process. To identify additional relevant literature, we also attempted to search Embase (detailed search string can be found in File S1) and retrieved appropriate articles from the reference lists of the articles included in the analysis from the initial selection.

### Study Selection

Studies were eligible for inclusion in this systematic review when they met the following criteria: (1) studies had to be situated in mainland China; (2) participants could not include the following high-risk populations: CSW and their clients, STI clinic attendees, men who have sex with men, commercial blood donors, and injecting drug users; (3) quantitative studies had to report on at least one of the following indicators of high-risk sexual behavior: premarital sex, commercial sex, multiple sex partners, condom use, or the prevalence of STIs; (4) studies had to be cross-sectional or longitudinal. For intervention studies, pre-intervention data was also eligible; (5) they had to be peer-reviewed, empirical studies, published in Chinese or English. Engaging in commercial sex was referred to as either having paid or been paid for sex. The prevalence of STIs was the proportion of participants that had chlamydia, syphilis, or gonorrhea (three common, reportable STIs in China), which was diagnosed based on laboratory testing. When two or more articles shared the same study data or were published in both English and Chinese sources, we excluded the articles published earlier or the ones published in the Chinese language. We did not include published surveillance data that only included information about the prevalence of HIV from the local or central government, because HIV is a chronic infection that cannot indicate recent sexual risk behaviors, but we did include studies on HIV incidence.

### Data Abstraction and Management

We extracted the following information from all eligible studies: (1) general information: first author, publication year, study location, and study year; for studies with no information on the study year, we assumed the study year was two years earlier than the publication year; when the study year concerned multiple years, we used the midpoint; (2) study design: sampling venues, methods, demographics (age, gender, and marital status), and laboratory testing methods; (3) indicators of sexual risk behaviors: proportions of participants engaging in premarital sex, commercial sex, or sex with multiple partners; condom use, and the prevalence of STIs. For study location, we categorized the 31 provinces/autonomous regions of mainland China into five modernization (development level) classes, according to China’s Modernization Report 2010 [16] as follows: (first class) provinces that accomplished 95%–100% modernization; (second class) provinces with 90%–94% modernization; (third class) provinces with 85%–89% modernization; (fourth class) provinces with 80%–84% modernization; and (fifth class) provinces with less than 80% modernization (see File S2). The development of provinces is unequal in China, and the modernization level indicates how developed the provinces or cities are as compared to Hong Kong. Studies that concern national or multicenter data (12 studies) are considered as a separate category. The proportions of participants that used condoms were assessed as “condom use at last intercourse” and “consistent condom use during the last month to a year”. Data about condom use with casual or commercial sex partners during the past one, six, twelve months was combined, because condom use in the past month to year is not so different (see Table S2 in File S3). The proportions of sexual behaviors and the prevalences of STIs were proportional to the number of responding participants of each study. We imported all the information into Excel and the eligible studies were clustered into three groups: (1) floating population; (2) college students; (3) other groups, including young, out-of-school individuals and community residents. An overview of the extracted information can be found in File S3 (Table S1–S3).

### Quality Assessment

We adjusted a validated quality assessment tool for cross-sectional studies [17] and used the following two criteria for studies of high quality: (1) sample size larger than 200, as participants in studies with small sample size may be not representative of the target population, (2) participants mean age range from 18 to 50 years old, as people in this age group are most sexually active. Two Chinese researchers evaluated study quality, and uncertainties were resolved by discussion with two other authors. Only studies with all the above two criteria were considered of high quality and included in the statistical analysis. Information regarding research methodology of reviewed studies can be found in File S3 (Table S1–S3).

### Statistical Analysis

We first plotted the indicators of sexual risk behaviors against the study year for each subgroup, i.e. floating population, college students, and other groups (out-of-school youth, community residents, and women from gynecological or obstetric clinics). The indicators were: proportions of participants that engaged in premarital sex, commercial sex, or sex with multiple partners, condom use, and the prevalence of STIs (i.e. chlamydia, syphilis, and gonorrhea). In the figures we visually separated overlapping data points for clarification, but the calculations were based on the actual study year.

To explore potential trends over the years, we used generalized linear mixed model (GLMM), which takes into account heterogeneity of the sample size and estimate effect of the various studies by including a random effect term. In each model, we included the factor ‘study year’, together with possible cofactors that may be confounders, and their interaction with ‘study year’. The cofactors included were: the modernization class of the province/autonomous region where the study was conducted (see Quality assessment above), sampling methods (0 = non-probability based sampling, 1 = probability based sampling), gender composition (1 = mainly females: male proportion <40%; 2 = females and males almost equal: male proportion: 40–60%; 3 = mainly males: male proportion >60%), age (0 = mainly young participants: mean age ≤30 years, 1 = mainly older participants: mean age >30 years), and marital status of the participants (1 = mainly unmarried participants: married proportion <40%; 2 = married and unmarried participants almost equal: married proportion: 40–60%; 3 = mainly married participants: married proportion >60%). When there was no significant factor retained, we used the univariate results from GLMM of indicators against study year (dashed lines) in the figures to illustrate the general overall pattern of the trends”.

## Results

### Study Identification and Selection

**Figure 1:**
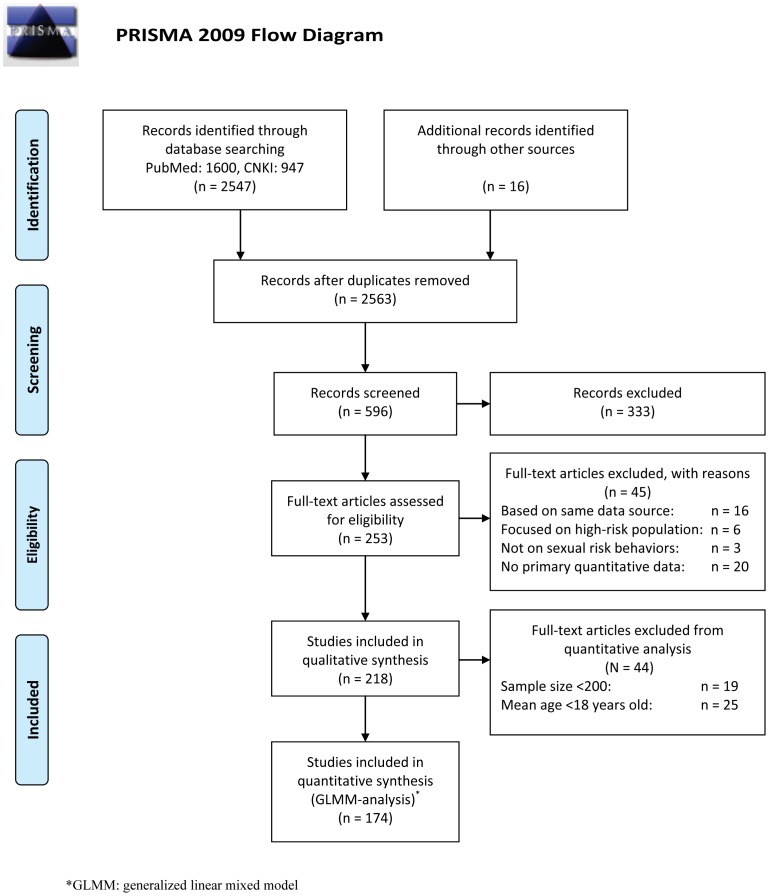
Flow chart of the literature selection process.

Our initial search strategy identified 2547 articles from the two electronic databases. After applying the selection criteria, the full texts of 247 articles were read. 16 additional articles were identified through the reference lists of the included articles from the initial selection and 174 were eligible in our final analysis (Table S1–S3 in File S3). The selection process is illustrated in Fig. 1. The 174 included studies assessed 932,931 participants in total, with male ratio ranging from 0–100% and age ranging from 14–74 years. Of the total 174 studies included, the majority had investigated the floating population and college students (55 and 73 studies, respectively). The remaining studies (n = 46) targeted other groups; in particular, the young, out-of-school population, community residents, and women recruited from gynecological or obstetric clinics.

### Study Characteristics

In the total of 174 studies, we could not identify studies conducted before 1999 that focused on the floating population, studies before 1995 that focused on college students, or studies before 1986 that focused on other groups. The included studies covered 25 of 31 provinces/autonomous regions in mainland China (no studies were identified that were conducted in Tibet, Qinghai, Hunan, Hainan, Xinjiang, and Ningxia provinces). The majority (78%, 43/55) of studies that focused on the floating population recruited their participants from routine venues; e.g., factories, restaurants, retail shops, and markets. All of the studies that focused on college students recruited their participants from campuses. Of the studies that focused on other groups, 76% (32/46) recruited participants from the general community. Most studies (86%, 149/174) used a probability-based sampling method; the remaining studies used convenience sampling or a quota sampling method. Furthermore, the majority of included studies only contained self-reported information. The prevalence of STIs was reported in only nine studies that focused on the floating population, two studies that focused on college students, and seventeen studies that focused on other groups. No study reported HIV incidence. Of these studies that reported STIs, blood samples were used to test syphilis and urinary samples were used to test chlamydia and gonorrhea. The majority (80%, 12/15) of studies reporting positive syphilis were based on confirmatory tests (TPPA or TPHA).

### Data Synthesis of Floating Population

**Figure 2:**
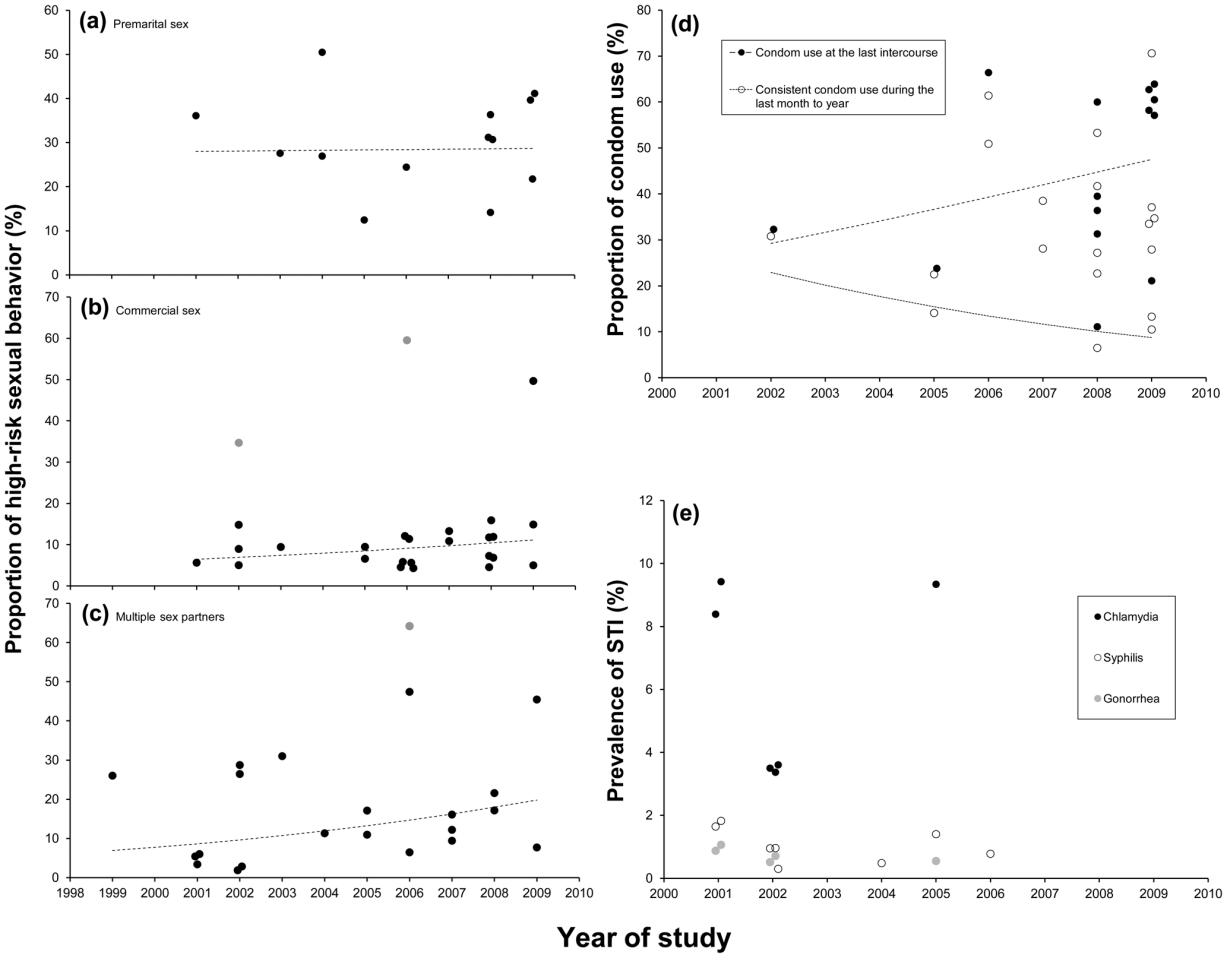
Trend analyses of indicators of high-risk sexual behavior in the floating population in China. (a) proportion that engaged in premarital sex; (b) proportion that engaged in commercial sex; (c) proportion that engaged in sex with multiple partners; (d) proportion that used condoms with casual or commercial sex partners; (e) prevalence of STIs. The dashed lines indicate the average trends calculated by generalized linear mixed model. Detailed information about the parameters of the dashed lines is given in File S4.

The majority of the included 55 studies that focused on the floating population used similar sampling methods and recruited participants from similar venues. They differed in the gender composition, the marital status, and the modernization class of study location. Of the nine studies reporting the prevalence of chlamydia, syphilis and/or gonorrhea, three reported only one STI, two reported two STIs and four reported all three STIs (see Table S3 in File S3). Two studies reported extremely high proportion of engaging in commercial sex (Fig. 2b) and having multiple sex partners (Fig. 2c) among migrant truck drivers. Among other migrants, we did not identify any significant trend of increased engagement in high-risk sexual behaviors (Fig. 2a–c). A slight increase across the years was found in recent condom use with casual partners/sex workers, but it was not significant (Fig. 2d).This result would most likely indicate a decreasing risk of STIs, however, the prevalence of STIs showed no particular trend over time (Fig. 2e). Results of the GLMM analysis can be found in File S4.

### Data Synthesis of College Students

**Figure 3:**
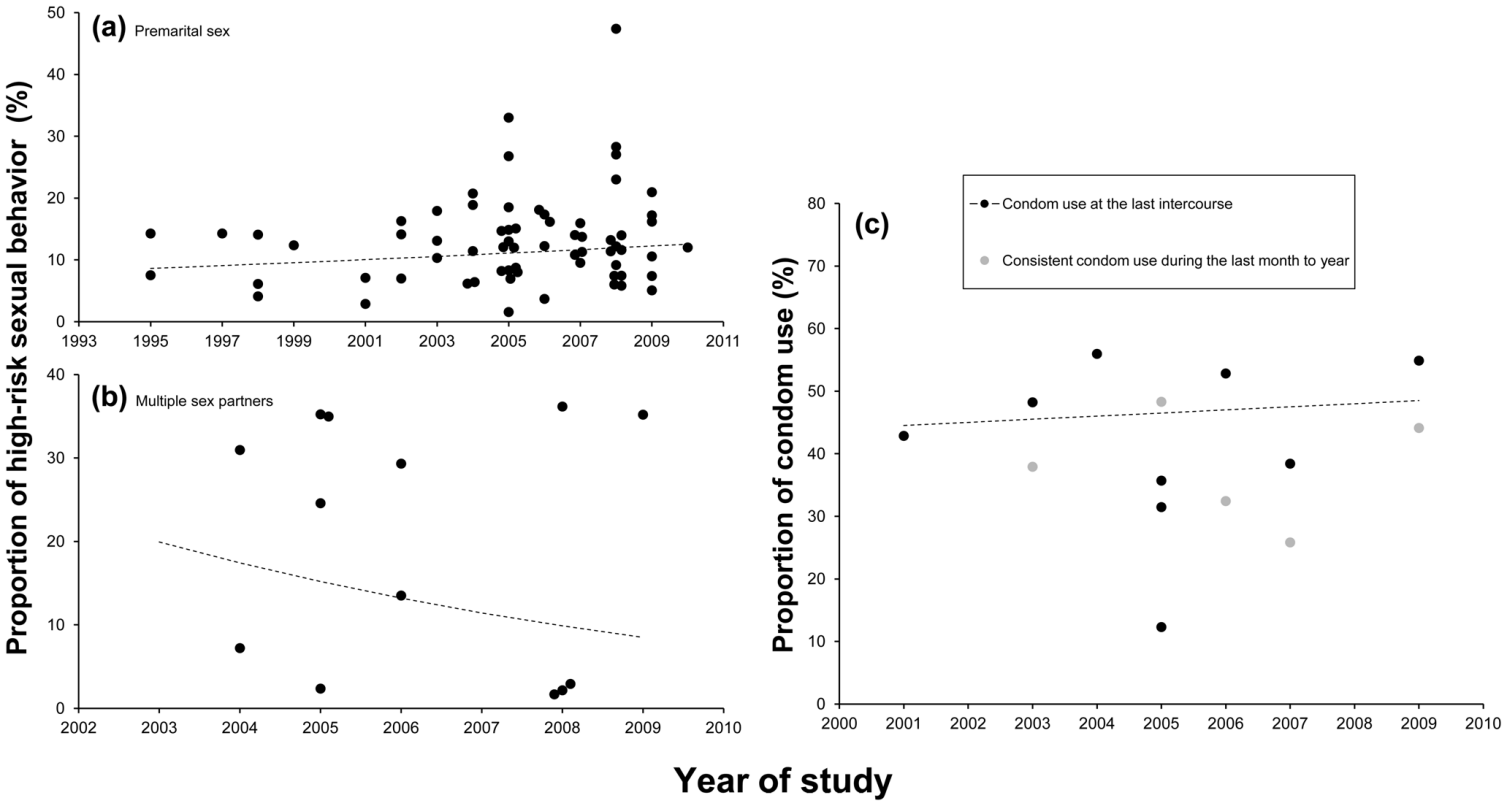
Trend analyses of indicators of high-risk sexual behavior in college student population in China. (a) proportion that engaged in premarital sex; (b) proportion that engaged in sex with multiple partners; (c) proportion that used condoms. The dashed lines indicate the average trends calculated by generalized linear mixed model. Detailed information about the parameters of the dashed lines is given in File S4. The grey dots (b & c) represent studies among migrant truck drivers that were not included in the average trend analysis.

Of the studies that reported sexual risk behaviors among college students, only four presented the percentage that engaged in commercial sex, and that percentage varied widely (range 0–11%; average 5·6%; data not shown) [18]–[21]. The majority of the included studies on college students reported the percentage that engaged in premarital sex or sex with multiple partners. A slightly increasing trend was observed only in the percentage that engaged in premarital sex (Fig. 3a). This increasing trend was not significant in the univariate GLMM analysis (see results in File S4), and not significant after adjusting for modernization class and gender (p = 0.20 and 0.99, respectively). Studies that investigated condom use among college students recruited participants from different universities and in different locations; however, all the included studies reported a similar proportion of students that used condoms both at last sexual intercourse and during last month to year (Fig. 3c). Two studies presented the prevalence of STIs (one for syphilis, the other for gonorrhea) among college students, and both found a very low prevalence. In one study, none of the sampled college students was infected with syphilis [19]; in the other study, the prevalence of gonorrhea was 0·4% [22].

### Data Synthesis of other, more General, Population Groups

**Figure 4:**
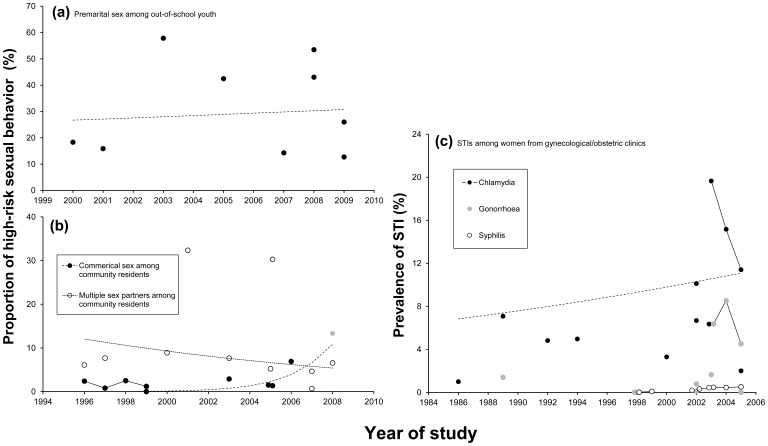
Trend analyses of indicators of high-risk sexual behavior in young, out-of-school individuals and community residents in China. (a) proportion that engaged in commercial sex among the community residents; (b) proportion that engaged in sex with multiple partners among community residents; (c) proportion that engaged in premarital sex among the out-of-school youth; (d) prevalence of STIs. The connected dots indicate longitudinal studies. The dashed lines indicate the average trends across the years. Detailed information about the parameters of the dashed lines was given in File S4. The grey dot (b) represents an outlier study that was not included in the average trend analysis.

The studies that reported information about sexual risk behaviors and condom use in other groups included out-of-school youth and community residents. No increased trends were observed in the reported proportion of out-of-school youth that engaged in premarital sex (Fig. 4a) or in the reported proportions of community residents that engaged in commercial sex or sex with multiple partners (Fig. 4b). Of the studies that investigated commercial sex among community residents, one 4-year longitudinal study was included. That study also reported that the proportion (0·8–2·4%) of residents that engaged in commercial sex was relatively stable over the years [23]. However, another study showed extreme results among older individuals (above 50 years old) in Yunnan; they reported a relatively high proportion (13·3%) of individuals had engaged in commercial sex [24]. Eighteen studies reported the prevalence of chlamydia, syphilis and/or gonorrhea, twelve reported one STI, four reported two kinds of STIs, and two studies reported all the three STIs (see Table S3 in File S3). The majority of the included studies that reported information about STIs were in clinical settings. The participants were predominately women recruited from gynecological or obstetric clinics and couples that had attended premarital medical check-ups. Among the women recruited from gynecological or obstetric clinics, the average prevalence of chlamydia appeared to have increased over time (Fig. 4c), but the only one longitudinal study on chlamydia revealed a strong decreased prevalence of chlamydia [25]. Three other studies revealed completely different patterns in the trend of syphilis prevalence: unclear, increasing and stable [26]–[28].

## Discussion

This is the first study that systematically reviewed literature on high-risk sexual behaviors and STI prevalence among population groups that do not belong to the classical high risk groups in China. Whereas other reviews identified an accelerating STIs/HIV epidemic among some of the classical high-risk populations (e.g. MSM) in China [5], [11], [12], our review did not reveal an increased tendency to engage in high-risk sexual behaviors. The available data does not indicate a clear trend of STIs in the general population groups included in our review.

Three important limitations of this study should be noted. Firstly, in the included studies, all the information collected on engaging in premarital sex, commercial sex, sex with multiple partners, and condom use was self-reported. This may have introduced a social desirability bias, particularly in China, where sex was, and remains (to some extent) to be, a taboo topic [2]. Moreover, the taboo was more relaxed in the later than in the earlier study years. Thus, participants in the later studies may have felt less inhibited, and thus, more likely to report the truth. In fact, if this had been taken into account, we may even have found a decreased tendency to engage in high-risk sexual behaviors over time.

Secondly, we identified only a few studies conducted before 2000. This was due to the fact that the Chinese government and academic organizations did not display high interest in studying sexual risk behaviors until the mid-1990s, when an outbreak of HIV occurred in the central provinces. Then, in the late 1990s, the international and national agencies began funding research on sexual risk behaviors and STIs. Consequently, our conclusion that there has been no increase in the tendency to engage in high-risk sexual behaviors has focused only on the past decade.

Thirdly, we only identified four longitudinal studies [23], [25]–[27].The majority of collected data was reported in cross-sectional studies, which may be less reliable for analyzing general trends, due to issues of comparability. Nevertheless, the four included longitudinal studies revealed no particular trend consistent with an increased tendency to engage in high-risk sexual behaviors. In some cases, they suggested the opposite tendency. For instance, one longitudinal study showed a clearly decreased prevalence of chlamydia among women recruited from gynecological or obstetric clinics [25].

We did not attempt Meta-regression because the objective of our systematic review was not to obtain a single summarized ‘effect estimate’ or to explore reasons for the heterogeneity of included studies in our systematic review. Nevertheless, different study designs (e.g. sampling method and demographic composition) could bias the significance of the factor ‘study year’. We therefore used GLMM based on the relatively comparable category of population groups, and integrated cofactors such as sampling method, age distribution, gender and marital composition in the analysis. We did not do any further subgroup analysis, as there is only study-level data and no individual data is available.

Our findings contrasted, or were at least inconsistent, with our expectations. We did not find any other increased tendencies to engage in sexual risk behaviors among Chinese population groups that do not belong to the classical high risk groups. This reassuring observation might be explained by the continuous efforts of the Chinese government and various non-government organizations to educate people about HIV/STIs through mass media and condom promotions. Thus, although attitudes in the Chinese population have become more tolerant toward engaging in premarital sex, commercial sex, casual sex, and sex with multiple partners, the knowledge and awareness of HIV/STIs may also have increased [29]. This knowledge may have counteracted the impact of the re-flourishing commercial sex industry and discouraged high-risk sexual behaviors. In addition, a change in the demographics of the sampled population over the years may partly explain the unexpected observations among the floating population. In the early period, the floating population comprised mainly single males, who may have sought sexual services; the later floating population included migrating families and ‘temporary couples’ [9]. These so-called temporary couples are single migrants who meet each other in the same working area and form a temporary fixed sexual partnership. Thus, the proportion that engaged in high-risk sexual behaviors might be expected to decrease.

Our results revealed no clear increasing trend of STIs from the available data among floating population and women from gynecological or obstetric clinics (Fig. 2e and 4c). This seems to conflict with the national surveillance data, which reveals a rapid increasing trend of syphilis among the whole population [11]. This phenomenon may be explained as follows: first, studies in our review focused on three population groups, who may not be very representative of the whole population. The discrepancy between our results and the surveillance data could have been caused by the paucity and diversity of the available data. Second, the increasing trend of syphilis among the general population may root from the increased prevalence of syphilis in the high-risk groups [30]. However, as an indicator to describe consequence of engaging in sexual risk behaviors, even an increasing trend of STIs not necessarily means an increasing trend of engaging in sexual risk behaviors. The increasing prevalence of STIs among high-risk groups could have caused an increase in prevalence among the general population through their sexual connections, even if the sexual risk behaviors among the general population stayed stable. Also, the discrepancy between our results and the national surveillance data concerns only the trend for syphilis. The surveillance data did not show an increasing trend for other STIs, such as gonorrhea [11], which is consistent with our results.

We reiterate that the aim of this systematic review is to investigate possible trends of engaging in high-risk sexual behaviors, and not of studying the percentage/prevalence values of STIs. The three population groups in our review may not be completely representative of the general population. Comparing percentages of engaging in high-risk sexual behaviors and prevalence of STIs between different population subgroups, or making inference of the percentage/prevalence values of STIs among the general population as a whole, should be avoided.

In conclusion, this systematic review revealed no clear increased tendency to engage in high-risk sexual behaviors, with an unclear trend pattern of STIs, among general Chinese population groups not belonging to the classical high risk groups. This may very well reflect that the sexual risk behavior of the whole general population has not increased substantially. In response to the growing STIs/HIV epidemic, the Chinese government, together with a number of international organizations, actively initiated various intervention programs, such as China-UK AIDS projects, Global Funds rounds 3 to 6 [31]. These programs target not only populations at highest risk, but also other population groups (e.g. floating population) [31]. Because China remains a resource-limited country, it would be problematic to find that the general population had also increased their participation in high-risk sexual behaviors. If so, the STIs/HIV epidemic will be very difficult to stop and much more resources will be needed. Our results suggest that the STIs/HIV epidemic among the general population is still under control. We recommend China prioritizes the well-known high-risk groups, and avoids diluting the effect of available resources for prevention by spreading these thinly over the whole population, because the epidemic is unlikely to spread rapidly among the general population. On the other hand, these potential risk groups should not be neglected altogether. Even with a stable trend of engaging in sexual risk behaviors, STIs/HIV can continue to spread if all prevention and treatment activities are stopped.

## Supporting Information

File S1 Search strategy.
(DOCX)

File S2 Categorization of modernization classes.
(DOCX)

File S3 Table S1, Studies reporting sexual risk behaviors among population groups that do not belong to the classical high risk groups in China. Table S2, Studies reporting condom usage among population groups that do not belong to the classical high risk groups in China. Table S3, Studies reporting STIs among population groups that do not belong to the classical high risk groups in China.
(DOCX)

File S4 Results of generalized linear mixed model analysis (GLMM).
(DOCX)

Checklist S1 PRISMA Checklist for systematic reviews.
(PDF)
