# Supplementary material for: Trends in High-Risk Sexual Behaviors among General Population Groups in China: A Systematic Review
Source: PLoS One. 2013 Nov 13;8(11):e79320. doi: 10.1371/journal.pone.0079320 (PMC3827370; doi:10.1371/journal.pone.0079320)
Supplement: File S1 — Search strategy. (DOCX) [file pone.0079320.s001.docx]

**Supplementary materials**

**File S1: Search Strategy**

We searched Pubmed and the Chinese National Knowledge Infrastructure (CNKI) to identify relevant publications. The search terms included ‘sexual behavior’, ‘condoms’, ‘sexually transmitted diseases’, and ‘China’. Detailed search strings for Pubmed are as follows:

(((sexual behavior[Mesh] OR sexualit*[tw] OR sex[tw]) AND (Risk tak*[tw] OR Risk behav*[tiab] OR risky[tiab]))

OR

(condoms[tw] AND (absen*[tiab] OR without[tiab] OR "not used"[tiab] OR "not using"[tiab]) OR unsafe Sex[tw])

OR

((prostitut*[tiab] OR commercial sex[tiab]) )

OR

multiple partner*[tiab] OR multiple sex partner*[tiab] OR Extramarital Relations*[tw] OR premarital sex*[tiab]

OR

((sexually transmitted diseases[Mesh] OR sexually transmitted infection[tiab] OR STI*[tiab] )

AND (prevalen*[tw] OR epidemi*[tw] OR occurrence*[tiab] OR inciden*))))

AND

China [tw]

We also attempted to identify additional literature from Embase, but no relevant study was added. Search strings for Embase are as follows:

((sexual behavior/exp AND 'high risk behavior'/exp OR (sex NEAR/6 Risk*):de,ab,ti)

OR

((condoms NEAR/6 (absen* OR without OR 'not used' OR 'not using')):de,ab,ti

OR

'unsafe Sex':de,ab,ti) OR (prostitut* OR 'commercial sex'):de,ab,ti OR ('multiple partners' OR 'multiple sex partners' OR (Extramarital NEXT/1 (Relation* OR sex)) OR 'premarital sex'):de,ab,ti

OR

(('sexually transmitted disease'/exp OR (('sexually transmitted' NEXT/3 infect*) OR STI*):de,ab,ti)

AND (prevalen* OR epidemi* OR occurrence*):de,ab,ti))

AND China:de,ab,ti
