# Supplementary material for: Trends in High-Risk Sexual Behaviors among General Population Groups in China: A Systematic Review
Source: PLoS One. 2013 Nov 13;8(11):e79320. doi: 10.1371/journal.pone.0079320 (PMC3827370; doi:10.1371/journal.pone.0079320)
Supplement: File S2 — Categorization of modernization classes. (DOCX) [file pone.0079320.s002.docx]

**Supplementary materials**

**File S2: Modernization classes**

We categorized the 31 provinces/autonomous regions of mainland China into five modernization (development level) classes, according to China’s Modernization Report 2010 [1] as follows: (first class) provinces/autonomous regions that accomplished 95%-100% modernization; (second class) provinces/autonomous regions with 90%-94% modernization; (third class) provinces/autonomous regions with 85%-89% modernization; (fourth class) provinces/autonomous regions with 80%-84% modernization; and (fifth class) provinces/autonomous regions with less than 80% modernization (see S2). The development of provinces/autonomous regions is unequal in China, and the modernization level indicates how developed the provinces or autonomous regions are as compared to Hong Kong.

First class: Beijing, Shanghai, Tianjin, Zhejiang, Guangdong, Jiangsu

Second class: Fujian, Liaoning, Shandong, Chongqing, Hubei

Third class: Jilin, Inter Mongolia, Heilongjiang, Shanxi, Hebei, Ningxia, Shanxi, Anhui

Fourth class: Hunan, Jiangxi, Xinjiang, Gansu, Sichuan, Qinghai, Henan, Hainan

Fifth class: Guangxi, Tibet, Yunnan, Guizhou

**Reference**

1. China Development Gateway (2011) China's modernization report 2010. Economic issues
