# Supplementary material for: Trends in High-Risk Sexual Behaviors among General Population Groups in China: A Systematic Review
Source: PLoS One. 2013 Nov 13;8(11):e79320. doi: 10.1371/journal.pone.0079320 (PMC3827370; doi:10.1371/journal.pone.0079320)
Supplement: File S3 — Table S1, Studies reporting sexual risk behaviors among population groups that do not belong to the classical high risk groups in China. Table S2, Studies reporting condom usage among population groups that do not belong to the classical high risk groups in China. Table S3, Studies reporting STIs among population groups that do not belong to the classical high risk groups in China. (DOCX) [file pone.0079320.s003.docx]

**Table S1: Studies reporting sexual risk behaviors among population groups that do not belong to the classical high risk groups in China.** Each section (row in grey) represents different study population groups.

| **First author, publication year** | **Study design** | | | | | | | | **Sexual risk behaviors** | | | | | | | | | | | |
| --- | --- | --- | --- | --- | --- | --- | --- | --- | --- | --- | --- | --- | --- | --- | --- | --- | --- | --- | --- | --- |
|  | **Study year** | | **Study location** | **Study base*** | **Sample method#** | **Male(%)** | **Age range (Mean)** | **Marital status¶** | **Time span** | **Premarital sex** | | | | **Commercial sex** | | | **Multiple sex partners** | | | |
|  |  |  |  |  |  |  |  |  |  | **n** | **N** | **%** | | **n** | **N** | **%** | **n** | **N** | **%** | |
| ***Floating population*** | | |  |  |  |  |  |  |  |  |  |  | |  |  |  |  |  |  | |
| Anderson, 2003 [[1](#_ENREF_1)] | | 1999 | Beijing, Shanghai | IP, RW | CS | 72 | < 40: 80% | − | Lifetime | − | − | − | | − | − | − | 115 | 442 | 26.0 | |
| Detels, 2003[[2](#_ENREF_2)] | | 2001 | Fujian | RW | PBS | 48 | 18-40 | M/C:78% | Last year | − | − | − | | − | − | − | 79 | 1316 | 6.0 | |
| Li, 2009 [[3](#_ENREF_3)] | | 2001 | Fujian | RW | PBS | 48 | 18-40 | M/C:78% | Last 6 months | − | − | − | | − | − | − | 244 | 4510 | 5.4 | |
| NIMH, 2007 [[4](#_ENREF_4)] | | 2001 | Fujian | RW | PBS | 48 | 18-40 | M/C:78% | Last year | 122 | 338 | 36.1 | | 85 | 1508 | 5.6 | 51 | 1508 | 3.4 | |
| Hu, 2004 [[5](#_ENREF_5)] | | 2002 | Anhui | RW | PBS | 49 | 25-44: 75% | M: 84% | Last year | − | − | − | | − | − | − | 13 | 694 | 1.9 | |
| Li, 2004 [[6](#_ENREF_6)] | | 2002 | Beijing | RW | QS | 66 | 18-30 (25) | NM:48% | Lifetime | − | − | − | | 89 | 992 | 9.0 | 285 | 992 | 28.7 | |
|  | |  | Jiangsu | RW | QS | 66 | 18-30 (26) | NM:41% | Lifetime | − | − | − | | 172 | 1161 | 14.8 | 307 | 1161 | 26.4 | |
| Lou, 2005 [[7](#_ENREF_7)] | | 2002 | Shanghai | RW, CR | PBS | 45 | 15-24 | M/C:26% | Last year | 247 | 1092 | 22.6 | | 55 | 1092 | 5.0 | 31 | 1092 | 2.8 | |
| Sun, 2004 [[8](#_ENREF_8)] | | 2002 | Sichuan | TD | PBS | − | − | M: 90% | Last year | − | − | − | | 266 | 767 | 34.7 | − | − | − | |
| Lin, 2006 [[9](#_ENREF_9)] | | 2003 | Beijing | RW | QS | − | 18-30 | NM:77% | Lifetime | 607 | 2201 | 27.6 | | 85 | 900 | 9.4 | 279 | 900 | 31.0 | |
| Xie, 2006 [[10](#_ENREF_10)] | | 2004 | Guangdong | CR | PBS | 0 | 15-49 (30.6) | M: 100% | Lifetime | 1482 | 2936 | 50.5 | | − | − | − | 332 | 2936 | 11.3 | |
| Zhao, 2006 [[11](#_ENREF_11)] | | 2004 | Guangdong | RW | CS | 48 | 15-34 (20.6) | NM:100% | Lifetime | 359 | 1333 | 26.9 | | − | − | − | − | − | − | |
| Xiao, 2007 [[12](#_ENREF_12)] | | 2005 | Guangdong | RW | CS | 47 | 20-26:50% | NM:63% | Last year | − | − | − | | 11 | 167 | 6.6 | 39 | 228 | 17.1 | |
| Zhang, 2007 [[13](#_ENREF_13)] | | 2005 | Guangdong | RW | PBS | 0 | >15(24) | NM:100% | Last 6 months | 233 | 1872 | 12.4 | | − | − | − | 97 | 886 | 10.9 | |
| Zhao, 2005 [[14](#_ENREF_14)] | | 2005 | Yunnan | RW | CS | 100 | 15-51 (28.4) | M: 70% | Last year | − | − | − | | 22 | 232 | 9.5 | − | − | − | |
| Gao, 2010 [[15](#_ENREF_15)] | | 2006 | Yunnan | RW | PBS | 100 | 17-64 (32) | M: 63% | Last year | − | − | − | 116 | | 959 | 12.1 | − | − | − |  |
| Li, 2007 [[16](#_ENREF_16)] | | 2006 | Guangdong | TD | PBS | 100 | 21-62 (36) | M/C:82% | Last year | − | − | − | 153 | | 257 | 59.5 | 165 | 257 | 64.2 | |
| Li, 2007 [[17](#_ENREF_17)] | | 2006 | 8 provinces | RW | PBS | 66 | 18-40 (29) | M: 72% | Last month | − | − | − | 62 | | 544 | 11.4 | − | − | − | |
| Liu, 2007 [[18](#_ENREF_18)] | | 2006 | Jiangxi | MR | PBS | 62 | 16-52 (25.6) | NM:60% | Lifetime | 53 | 217 | 24.4 | 9 | | 160 | 5.6 | 14 | 217 | 6.5 |  |

**Table S1: Studies reporting sexual risk behaviors among population groups that do not belong to the classical high risk groups in China** *(continued)***.**

| **First author, publication year** | | **Study design** | | | | | | | **Sexual risk behaviors** | | | | | | | | | | |
| --- | --- | --- | --- | --- | --- | --- | --- | --- | --- | --- | --- | --- | --- | --- | --- | --- | --- | --- | --- |
|  |  | **Study year** | **Study location** | **Study base*** | **Sample method#** | **Male(%)** | **Age range (Mean)** | **Marital status¶** | **Time span** | **Premarital sex** | | | **Commercial sex** | | | | **Multiple sex partners** | | |
|  |  |  |  |  |  |  |  |  |  | **n** | **N** | **%** | **n** | **N** | **%** | | **n** | **N** | **%** |
| Li, 2008 [[19](#_ENREF_19)] | 2006 | | Mongolia | RW | PBS | 41 | 16-65 (33) | M/C:73% | Lifetime | − | − | − | 44 | 965 | 4.6 | − | | − | − |
| Li, 2010 [[20](#_ENREF_20)] | | 2006 | Guangdong | CR | PBS | 41 | 15-49 | M: 55% | Last 3 months | − | − | − | 35 | 812 | 4.3 | | − | − | − |
| Xin,2010 [[21](#_ENREF_21)] | | 2006 | Anhui | RW | PBS | 94 | 16-72 (39) | NM:20% | Last 6 months | − | − | − | 63 | 1082 | 5.8 | | − | − | − |
| He, 2009 [[22](#_ENREF_22)] | | 2007 | Shanghai | RW | QS | 44 | 18-34: 76% | M: 61% | Last year | − | − | − | − | − | − | | 193 | 2051 | 9.4 |
| He, 2009 [[23](#_ENREF_23)] | | 2007 | Shanghai | RW | CS | 100 | 18-65 (33) | M: 83% | Lifetime | − | − | − | − | − | − | | 144 | 895 | 16.1 |
| Li, 2009 [[24](#_ENREF_24)] | | 2007 | Chongqing | RW | PBS | 68 | 18-50 (34) | M: 70% | Lifetime | − | − | − | 41 | 377 | 10.9 | | − | − | − |
| Mantell, 2011[[25](#_ENREF_25)] | | 2007 | Jiangsu | RW | CS | 26 | 18-57 (23) | M: 22% | Last month | − | − | − | 71 | 534 | 13.3 | | 84 | 689 | 12.2 |
| Chen, 2010 [[26](#_ENREF_26)] | | 2008 | Sichuan | CR | CS | 43 | 20-40: 53% | M: 73% | Last year | − | − | − | 24 | 204 | 11.8 | | − | − | − |
| Gao, 2009 [[27](#_ENREF_27)] | | 2008 | Shandong | RW | CS | 43 | 15-24 (20) | NM:96% | Lifetime | 290 | 945 | 30.7 | − | − | − | | − | − | − |
| Huang, 2011 [[28](#_ENREF_28)] | | 2008 | Shanghai | RW | PBS | 90 | 20-74 (39) | M: 84% | Lifetime | 93 | 657 | 14.2 | 45 | 657 | 6.8 | | − | − | − |
| Li, 2012 [[29](#_ENREF_29)] | | 2008 | Shanxi | MR | PBS | 53 | 26-45: 56% | M: 88% | Lifetime | 267 | 735 | 36.3 | − | − | − | | 126 | 735 | 17.1 |
| Lin, 2010 [[30](#_ENREF_30)] | | 2008 | Guangdong | RW | PBS | 41 | 20-30: 60% | NM:40% | Last 6 months | − | − | − | 25 | 210 | 11.9 | | − | − | − |
| Ren, 2011 [[31](#_ENREF_31)] | | 2008 | Shanghai | RW | PBS | 100 | 16-71 (39) | M: 81% | Lifetime | 468 | 1501 | 31.2 | 128 | 1757 | 7.3 | | 379 | 1757 | 21.6 |
| Tan, 2011 [[32](#_ENREF_32)] | | 2008 | Guangxi | RW | PBS | 89 | 18-48 (32) | M: 51% | Lifetime | − | − | − | 33 | 725 | 4.6 | | − | − | − |
| Zhang, 2010 [[33](#_ENREF_33)] | | 2008 | Sichuan | IP | CS | 85 | 17-66 (35) | M/C:86% | Last year | − | − | − | 81 | 509 | 15.9 | | − | − | − |
| Li, 2011 [[34](#_ENREF_34)] | | 2009 | Anhui | RW | PBS | 60 | 20-50: 79% | M: 58% | Last year | 281 | 683 | 41.1 | 83 | 1657 | 5.0 | | − | − | − |
| Wei, 2010 [[35](#_ENREF_35)] | | 2009 | Chongqing | RW | PBS | 73 | 18-46 (35) | M/C:84% | Last year | − | − | − | 185 | 1243 | 14.9 | | − | − | − |
| Zhang, 2010 [[36](#_ENREF_36)] | | 2009 | Guangdong | RW | PBS | 51 | 18-30 | M: 33% | Lifetime | 67 | 169 | 39.6 | − | − | − | | − | − | − |
| Zhang, 2011 [[37](#_ENREF_37)] | | 2009 | Shandong | RW | PBS | 0 | 18-29 (21) | NM:100% | Last 6 months | 177 | 814 | 21.7 | − | − | − | | 10 | 130 | 7.7 |
| Zuo, 2011 [[38](#_ENREF_38)] | | 2009 | Guangdong | RW | PBS | 100 | 18-59 (34) | NM:28% | Lifetime | − | − | − | 377 | 759 | 49.7 | | − | − | − |
|  | |  |  |  |  |  |  |  | Last year | − | − | − | − | − | − | | 345 | 759 | 45.5 |

**Table S1: Studies reporting sexual risk behaviors among population groups that do not belong to the classical high risk groups in China** *(continued)***.**

| **First author, publication year** | **Study design** | | | | | | | | | | | | | **Sexual risk behaviors** | | | | | | | | | | | | | | | | | | | |  |
| --- | --- | --- | --- | --- | --- | --- | --- | --- | --- | --- | --- | --- | --- | --- | --- | --- | --- | --- | --- | --- | --- | --- | --- | --- | --- | --- | --- | --- | --- | --- | --- | --- | --- | --- |
|  | **Study year** | **Study location** | | **Study base*** | | **Sample method#** | | **Male(%)** | | **Age range (Mean)** | | **Marital status¶** | | **Time span** | | **Premarital sex** | | | | | | **Commercial sex** | | | | | | **Multiple sex partners** | | | | | |  |
|  |  |  |  |  |  |  |  |  |  |  |  |  |  |  |  | **n** | | **N** | | **%** | | **n** | | **N** | | **%** | | **n** | | **N** | | **%** | |  |
| ***College students*** |  | |  | |  | |  | |  | |  | |  | |  | |  | |  | |  | |  | |  | |  | |  | |  | |  | |
| He, 1997 [[39](#_ENREF_39)] | 1995 | | Yunnan | | 3 U | | PBS | | 53 | | 17-27 (20) | | − | | Lifetime | | 322 | | 2257 | | 14.3 | | − | | − | | − | | − | | − | | − | |
| Wu, 1997 [[40](#_ENREF_40)] | 1995 | | Shanghai | | 2 U | | PBS | | 54 | | − | | − | | Lifetime | | 73 | | 971 | | 7.5 | | − | | − | | − | | − | | − | | − | |
| Li, 1999 [[41](#_ENREF_41)] | 1997 | | Beijing | | 5 U | | PBS | | 63 | | − | | − | | Lifetime | | 187 | | 1310 | | 14.3 | | 26 | | 1310 | | 2.0 | | − | | − | | − | |
| Tao, 1999 [[42](#_ENREF_42)] | 1998 | | Anhui | | 3 U | | PBS | | 72 | | − | | − | | Lifetime | | 54 | | 884 | | 6.1 | | − | | − | | − | | − | | − | | − | |
| Wang, 2000 [[43](#_ENREF_43)] | 1998 | | Shanghai | | 8 U | | PBS | | − | | − | | − | | Lifetime | | 122 | | 2974 | | 4.1 | | − | | − | | − | | − | | − | | − | |
| Xia, 2000 [[44](#_ENREF_44)] | 1998 | | Guangdong | | 8 U | | PBS | | 44 | | − | | − | | Lifetime | | 160 | | 1136 | | 14.1 | | − | | − | | − | | − | | − | | − | |
| Zhang, 2001 [[45](#_ENREF_45)] | 1999 | | Beijing | | 1 U | | PBS | | 49 | | 17-26 (21) | | − | | Lifetime | | 222 | | 1796 | | 12.4 | | − | | − | | − | | − | | − | | − | |
| Cottrell, 2004 [[46](#_ENREF_46)] | 2001 | | Jiangsu | | 19 U | | PBS | | − | | − | | − | | Lifetime | | 133 | | 1874 | | 7.1 | | − | | − | | − | | − | | − | | − | |
| Xiang, 2003 [[47](#_ENREF_47)] | 2001 | | Guangdong | | 5 U | | PBS | | 53 | | − | | − | | Lifetime | | 20 | | 694 | | 2.9 | | − | | − | | − | | − | | − | | − | |
| Yang, 2010 [[48](#_ENREF_48)] | 2001 | | 4 provinces | | − | | PBS | | − | | − | | − | | Lifetime | | 786 | | 11562 | | 6.8 | | − | | − | | − | | − | | − | | − | |
| Fan, 2004 [[49](#_ENREF_49)] | 2002 | | Guangdong | | 1 U | | PBS | | 46 | | − | | − | | Lifetime | | 63 | | 387 | | 16.3 | | − | | − | | − | | − | | − | | − | |
| Huang, 2005 [[50](#_ENREF_50)] | 2002 | | Hunan | | 6 U | | CS | | 68 | | 17-28 (20) | | − | | Lifetime | | 167 | | 1182 | | 14.1 | | − | | − | | − | | − | | − | | − | |
| Song, 2003 [[51](#_ENREF_51)] | 2002 | | Anhui | | 1 U | | PBS | | 61 | | 16-30 (20) | | − | | Lifetime | | 101 | | 1445 | | 7.0 | | − | | − | | − | | − | | − | | − | |
| Lin, 2009 [[52](#_ENREF_52)] | 2003 | | Guangdong | | 1 U | | PBS | | 44 | | − | | − | | Lifetime | | 552 | | 3080 | | 17.9 | | − | | − | | − | | − | | − | | − | |
| Dong, 2005 [[53](#_ENREF_53)] | 2003 | | Jilin | | 1 U | | PBS | | 37 | | − | | − | | Lifetime | | 115 | | 1115 | | 10.3 | | − | | − | | − | | − | | − | | − | |
| Ma, 2006 [[54](#_ENREF_54)] | 2003 | | Zhejiang | | 2 U | | PBS | | 50 | | 20-23 (20) | | − | | Last year | | 2965 | | 22646 | | 13.1 | | − | | − | | − | | 322 | | 2015 | | 16.0 | |
| Chen, 2005 [[55](#_ENREF_55)] | 2004 | | Fujian | | 8 U | | PBS | | 43 | | − | | − | | Lifetime | | 302 | | 4709 | | 6.4 | | − | | − | | − | | − | | − | | − | |
| Guan, 2006 [[56](#_ENREF_56)] | 2004 | | Liaoning | | 9 U | | PBS | | − | | − | | − | | Lifetime | | 186 | | 3018 | | 6.2 | | − | | − | | − | | − | | − | | − | |
| Lonn, 2007 [[57](#_ENREF_57)] | 2004 | | Xinjiang | | 1 U | | PBS | | 39 | | 17-24 (21) | | − | | Lifetime | | 73 | | 352 | | 20.7 | | − | | − | | − | | − | | − | | − | |
| Sun, 2006 [[58](#_ENREF_58)] | 2004 | | Sichuan | | 5 U | | PBS | | 37 | | − | | − | | Lifetime | | 348 | | 1843 | | 18.9 | | − | | − | | − | | 133 | | 1843 | | 7.2 | |

**Table S1: Studies reporting sexual risk behaviors among population groups that do not belong to the classical high risk groups in China** *(continued)***.**

| **First author, publication year** | **Study design** | | | | | | | | **Sexual risk behaviors** | | | | | | | | | |
| --- | --- | --- | --- | --- | --- | --- | --- | --- | --- | --- | --- | --- | --- | --- | --- | --- | --- | --- |
|  | **Study year** | | **Study location** | **Study base*** | **Sample method#** | **Male(%)** | **Age range (Mean)** | **Marital status¶** | **Time span** | **Premarital sex** | | | **Commercial sex** | | | **Multiple sex partners** | | |
|  |  |  |  |  |  |  |  |  |  | **n** | **N** | **%** | **n** | **N** | **%** | **n** | **N** | **%** |
| Yang, 2010 [[48](#_ENREF_48)] | | 2004 | 4 provinces | − | PBS | 42 | − | − | Lifetime | 668 | 10045 | 6.7 | − | − | − | − | − | − |
| Zhai, 2007 [[59](#_ENREF_59)] | | 2004 | Beijing | 2 U | PBS | 42 | − | − | Lifetime | 130 | 1138 | 11.4 | − | − | − | − | − | − |
| Cai, 2006 [[60](#_ENREF_60)] | | 2005 | Anhui | 1 U | PBS | 57 | − | − | Lifetime | 148 | 1227 | 12.1 | − | − | − | 29 | 1227 | 2.4 |
| Chen, 2008 [[61](#_ENREF_61)] | | 2005 | Shanghai | 14 U | PBS | 50 | 15-34 | − | Lifetime | 764 | 5067 | 15.1 | − | − | − | − | − | − |
| Kong, 2007 [[62](#_ENREF_62)] | | 2005 | Beijing | 6 U | PBS | 54 | 20-26 | − | Lifetime | 77 | 416 | 18.5 | − | − | − | − | − | − |
| Li, 2007 [[63](#_ENREF_63)] | | 2005 | Guangdong | 1 U | PBS | 50 | 18-23 (21) | − | Lifetime | 136 | 508 | 26.8 | − | − | − | 31 | 88 | 35.2 |
| Li, 2007 [[64](#_ENREF_64)] | | 2005 | Hubei | 1 U | PBS | 0 | 16-23 (20) | − | Lifetime | 42 | 286 | 14.7 | − | − | − | 13 | 42 | 31.0 |
| Li, 2007 [[65](#_ENREF_65)] | | 2005 | Sichuan | 1 U | PBS | 51 | 17-35 (20) | − | Lifetime | 40 | 499 | 8.0 | − | − | − | − | − | − |
| Sun, 2007 [[66](#_ENREF_66)] | | 2005 | Beijing | 2 U | PBS | 36 | 18-24 | − | Lifetime | 88 | 1059 | 8.3 | − | − | − | − | − | − |
| Song, 2010 [[67](#_ENREF_67)] | | 2005 | Nationwide | 84 U | PBS | 47 | 16-24 (20) | − | Lifetime | 3803 | 33653 | 11.3 | − | − | − | − | − | − |
| Tan, 2007 [[68](#_ENREF_68)] | | 2005 | Hubei | 1 U | PBS | 49 | − | − | Lifetime | 18 | 259 | 6.9 | 0 | 259 | 0.0 | − | − | − |
| Xu, 2007 [[69](#_ENREF_69)] | | 2005 | Hubei | 5 U | PBS | 0 | 16-26 (21) | − | Lifetime | 118 | 985 | 12.0 | − | − | − | 29 | 118 | 24.6 |
| Xu, 2007 [[70](#_ENREF_70)] | | 2005 | Jiangxi | 1 U | PBS | 42 | − | − | Lifetime | 11 | 701 | 1.6 | − | − | − | − | − | − |
| Xu, 2007 [[71](#_ENREF_71)] | | 2005 | Guangxi | 4 U | PBS | 45 | 15-29 (21) | − | Lifetime | 176 | 2015 | 8.7 | − | − | − | 50 | 143 | 35.0 |
| Zeng, 2007 [[72](#_ENREF_72)] | | 2005 | Guangdong | 2 U | PBS | 64 | 16-24 | − | Lifetime | 185 | 1245 | 14.9 | − | − | − | − | − | − |
| Zhang, 2007 [[73](#_ENREF_73)] | | 2005 | Sichuan | 2 U | PBS | 0 | 16-28 (21) | − | Lifetime | 533 | 1615 | 33.0 | − | − | − | − | − | − |
| Zhao, 2006 [[74](#_ENREF_74)] | | 2005 | Hebei | 1 U | PBS | − | − | − | Lifetime | 260 | 2000 | 13.0 | − | − | − | − | − | − |
| Zhou, 2006 [[75](#_ENREF_75)] | | 2005 | Beijing | 2 U | PBS | 49 | − | − | Lifetime | 42 | 512 | 8.2 | − | − | − | − | − | − |
| Liu, 2007 [[76](#_ENREF_76)] | | 2006 | Hebei | 3 U | PBS | 52 | 19-22 | − | Lifetime | 36 | 976 | 3.7 | − | − | − | − | − | − |
| Wu, 2007 [[77](#_ENREF_77)] | | 2006 | Zhejiang | 1 U | PBS | 69 | (22) | − | Last year | 407 | 3326 | 12.2 | 45 | 407 | 11.1 | 55 | 407 | 13.5 |
| Wang, 2007 [[78](#_ENREF_78)] | | 2006 | Hubei | 1 U | PBS | 70 | 17-26 (21) | − | Lifetime | 127 | 786 | 16.2 | − | − | − | − | − | − |

**Table S1: Studies reporting sexual risk behaviors among population groups that do not belong to the classical high risk groups in China** *(continued)***.**

| **First author, publication year** | **Study design** | | | | | | | | **Sexual risk behaviors** | | | | | | | | | |
| --- | --- | --- | --- | --- | --- | --- | --- | --- | --- | --- | --- | --- | --- | --- | --- | --- | --- | --- |
|  | **Study year** | | **Study location** | **Study base*** | **Sample method#** | **Male(%)** | **Age range (Mean)** | **Marital status¶** | **Time span** | **Premarital sex** | | | **Commercial sex** | | | **Multiple sex partners** | | |
|  |  |  |  |  |  |  |  |  |  | **n** | **N** | **%** | **n** | **N** | **%** | **n** | **N** | **%** |
| Sun, 2010 [[79](#_ENREF_79)] | 2006 | | 9 provinces | 30 U | PBS | 49 | 21 | − | Lifetime | 1707 | 18795 | 9.1 | − | − | − | − | − | − |
| Wang, 2010 [[80](#_ENREF_80)] | 2006 | | 6 provinces | − | − | − | − | − | Lifetime | 191 | 4524 | 4.3 | − | − | − | − | − | − |
| Xu, 2011 [[81](#_ENREF_81)] | | 2006 | Hubei | 13 U | PBS | 0 | 17-26 (20) | − | Lifetime | 323 | 1862 | 17.3 | − | − | − | − | − | − |
| Yan, 2009 [[82](#_ENREF_82)] | | 2006 | Hubei | 16 U | PBS | 0 | 16-27 (20) | NM:100% | Lifetime | 863 | 4769 | 18.1 | − | − | − | 253 | 863 | 29.3 |
| Liu, 2009 [[83](#_ENREF_83)] | | 2007 | Zhejiang | 1 U | PBS | 27 | − | − | Lifetime | 76 | 477 | 15.9 | − | − | − | − | − | − |
| Peng, 2009 [[84](#_ENREF_84)] | | 2007 | Beijing | 6 U | PBS | 52 | 17-26 (20) | − | Lifetime | 158 | 1151 | 13.7 | − | − | − | − | − | − |
| Wang, 2009 [[85](#_ENREF_85)] | | 2007 | Zhejiang | 2 U | PBS | 44 | 17-26 (21) | − | Lifetime | 30 | 315 | 9.5 | − | − | − | − | − | − |
| Cheng, 2010 [[86](#_ENREF_86)] | | 2007 | Shanghai | CR | PBS | 45 | − | − | Lifetime | 223 | 2062 | 10.8 | − | − | − | − | − | − |
| Duan, 2011 [[87](#_ENREF_87)] | | 2007 | Shanxi | 1 U | PBS | 45 | − | − | Lifetime | 257 | 2276 | 11.3 | − | − | − | − | − | − |
| Yang, 2010 [[48](#_ENREF_48)] | | 2007 | 4 provinces | − | PBS | 42 | − | − | Lifetime | 859 | 9824 | 8.7 | − | − | − | − | − | − |
| Zhou, 2009 [[88](#_ENREF_88)] | | 2007 | 7 provinces | 49 U | PBS | 47 | − | − | Lifetime | 10693 | 74258 | 14.4 | − | − | − | − | − | − |
| Zhou, 2010 [[89](#_ENREF_89)] | | 2007 | Jiangxi | 3 U | PBS | 51 | 17-24 (20) | − | Lifetime | 151 | 1077 | 14.0 | − | − | − | − | − | − |
| Zhou, 2011 [[90](#_ENREF_90)] | | 2007 | 7 provinces | 49 U | PBS | 47 | 18-24 | − | Lifetime | 7230 | 62326 | 11.6 | − | − | − | − | − | − |
| Chen, 2009 [[91](#_ENREF_91)] | | 2008 | Shanghai | 2 U | PBS | 59 | 19-22 | − | Lifetime | 143 | 1232 | 11.6 | − | − | − | − | − | − |
| Pan, 2009 [[92](#_ENREF_92)] | | 2008 | Zhejiang | 1 U | PBS | 60 | 18-23 | − | Lifetime | 104 | 744 | 14.0 | − | − | − | − | − | − |
| Albrektsson, 2009 [[93](#_ENREF_93)] | | 2008 | Hubei | 1 U | PBS | − | − | − | Lifetime | 56 | 611 | 9.2 | − | − | − | − | − | − |
| Wang, 2010 [[80](#_ENREF_80)] | | 2008 | 6 provinces | − | − | − | − | − | Lifetime | 265 | 4562 | 5.8 | − | − | − | − | − | − |
| Wu, 2009 [[94](#_ENREF_94)] | | 2008 | Shandong | 1 U | PBS | 45 | − | − | Lifetime | 35 | 601 | 5.8 | − | − | − | 13 | 601 | 2.2 |
| Zhang, 2009 [[95](#_ENREF_95)] | | 2008 | Liaoning | 2 U | PBS | 38 | − | − | Lifetime | 75 | 615 | 12.2 | − | − | − | 18 | 615 | 2.9 |
| Zhang, 2010 [[96](#_ENREF_96)] | | 2008 | Chongqing | 3 U | PBS | 35 | − | − | Lifetime | 71 | 954 | 7.4 | − | − | − | 16 | 954 | 1.7 |

**Table S1: Studies reporting sexual risk behaviors among population groups that do not belong to the classical high risk groups in China** *(continued)***.**

| **First author, publication year** | **Study design** | | | | | | | | | | | | | | | | | | | | | | **Sexual risk behaviors** | | | | | | | | | | | | | | | | | | | | | | | | | | |
| --- | --- | --- | --- | --- | --- | --- | --- | --- | --- | --- | --- | --- | --- | --- | --- | --- | --- | --- | --- | --- | --- | --- | --- | --- | --- | --- | --- | --- | --- | --- | --- | --- | --- | --- | --- | --- | --- | --- | --- | --- | --- | --- | --- | --- | --- | --- | --- | --- | --- |
|  | **Study year** | | | | | **Study location** | | | | | | **Study base*** | **Sample method#** | **Male(%)** | | | | **Age range (Mean)** | | | **Marital status¶** | | **Time span** | | **Premarital sex** | | | | | | | | | **Commercial sex** | | | | | | **Multiple sex partners** | | | | | | | | |  |
|  |  |  |  |  |  |  |  |  |  |  |  |  |  |  |  |  |  |  |  |  |  |  |  |  | **n** | | **N** | | **%** | | | | **n** | | **N** | | | **%** | **n** | | | | | **N** | | | | **%** |  |
| You, 2010 [[97](#_ENREF_97)] | | 2008 | | | Heilongjiang | | | | | | | 1 U | PBS | | | 55 | | 17-24 (22) | C: 14.5% | | | | Lifetime | | 228 | 843 | | | | | 27.0 | | − | | − | | | − | − | | − | | | | | | − | |  |
| You, 2010 [[98](#_ENREF_98)] | | 2008 | | | Heilongjiang | | | | | | | 3 U | PBS | | | 52 | | − | − | | | | Lifetime | | 295 | 1282 | | | | | 23.0 | | − | | − | | | − | − | | − | | | | | | − | |  |
| Wu, 2009 [[99](#_ENREF_99)] | 2008 | | | | | | Henan | | | | | 2 U | PBS | | | 44 | | − | − | | | | Lifetime | | 42 | | | 566 | 7.4 | | | | − | | − | | | − | − | | | | − | | | | − | |  |
| Zhu, 2009 [[100](#_ENREF_100)] | 2008 | | | | | | Gansu | | | | | 2 U | PBS | | | 40 | | − | − | | | | Lifetime | | 37 | | | 615 | 6.0 | | | | − | | − | | | − | − | | | | − | | | | − | |  |
| Yang, 2010 [[101](#_ENREF_101)] | 2008 | | | | | | Jiangxi | | | | | 5 U | PBS | | | 46 | | 18-24 | | NM:97% | | | Lifetime | | 1232 | | | 2600 | 47.4 | | | | − | | − | | | − | − | | | | − | | | | − | |  |
| Yang, 2010 [[102](#_ENREF_102)] | 2008 | | | | | | Sichuan | | | | | 8 U | PBS | | | 51 | | 17-24 (21) | | − | | | Lifetime | | 990 | | | 3500 | 28.3 | | | | − | | − | | | − | − | | | | − | | | | − | |  |
| Chen, 2010 [[103](#_ENREF_103)] | 2008 | | | | | | Guizhou | | | | | 1 U | PBS | | | 45 | | 17-26 (21) | | − | | | Lifetime | | 177 | | | 1554 | 11.4 | | | | − | | − | | | − | 64 | | | | 177 | | | | 36.2 | |  |
| Shi, 2010 [[104](#_ENREF_104)] | 2008 | | | | | | Guangxi | | | | | 2 U | PBS | | | − | | − | | − | | | Lifetime | | 77 | | | 583 | 13.2 | | | | − | | − | | | − | − | | | | − | | | | − | |  |
| Chen, 2011 [[105](#_ENREF_105)] | 2009 | | | | | | | | Guizhou | | 2 U | | PBS | | 44 | | | 17-24 | | | − | | Lifetime | | 162 | | 1534 | | 10.6 | | | | 15 | | 162 | | | 9.3 | 57 | | | | 162 | | | | 35.2 | |  |
| Wang, 2010 [[80](#_ENREF_80)] | 2009 | | | | | | | | 6 provinces | | − | | − | | − | | | − | | | − | | Lifetime | | 275 | | 4582 | | 6.0 | | | | − | | − | | | − | − | | | | − | | | | − | |  |
| Liu, 2011 [[106](#_ENREF_106)] | 2009 | | | | | | | | Jiangsu | | 7 U | | PBS | | 59 | | | 16-25 | | | − | | Lifetime | | 53 | | 717 | | 7.4 | | | | − | | − | | | − | − | | | | − | | | | − | |  |
| Wang, 2010 [[107](#_ENREF_107)] | 2009 | | | | | | | | Hubei | | 8 U | | PBS | | 66 | | | 17-25 (20) | | | − | | Lifetime | | 58 | | 1143 | | 5.1 | | | | − | | − | | | − | − | | | | − | | | | − | |  |
| Wang, 2011 [[108](#_ENREF_108)] | 2009 | | | | | | | | Chongqing | | 1 U | | PBS | | 100 | | | 17-24 (21) | | C: 0.5% | | Lifetime | | | 95 | | 552 | | 17.2 | | | | − | | − | | | − | − | | | | − | | | | − | |  |
| Yang, 2011 [[109](#_ENREF_109)] | 2009 | | | | | | | | Yunnan | | 7 U | | PBS | | 0 | | | 18-25 (22) | | − | | Lifetime | | | 124 | | 766 | | 16.2 | | | | − | | − | | | − | − | | | | − | | | | − | |  |
| Zhu, 2011 [[110](#_ENREF_110)] | 2009 | | | | | | | | Jilin | | 1 U | | PBS | | | | 0 | − | | − | | Lifetime | | | 449 | | 2143 | | 21.0 | | | | − | | − | | | − | − | | | | − | | | | − | |  |
| Guo, 2011 [[111](#_ENREF_111)] | 2010 | | | | | | | | Beijing | | 6 U | | PBS | | 48 | | | 16-28 (20) | | | − | | Lifetime | | 125 | | 1041 | | 12.0 | | | | − | | − | | | − | − | | | | − | | | | − | |  |
| ***Other groups*** | | | |  | | | | | |  | |  |  | | | |  |  | |  | |  | |  | | |  | | |  | |  | | | |  |  | |  | | |  | | |  | | | |  |
| Ma, 2001 [[112](#_ENREF_112)] | | | 1996 | | | | | Yunnan | | | | CR | PBS | | | | − | 15-69 | | M:59% | | Last month | | − | | | − | | | − | | | | − | | − | 2.4 | | − | | | − | | | | − | | |  |
| Sun, 2001 [[113](#_ENREF_113)] | | | 1996 | | | | | Jiangsu | | | | CR | PBS | | | | − | 18-30 | | − | | Lifetime | | − | | | − | | | − | | | | − | | − | − | | 33 | | | 541 | | | | 6.1 | | |  |

**Table S1: Studies reporting sexual risk behaviors among population groups that do not belong to the classical high risk groups in China** *(continued)***.**

| **First author, publication year** | **Study design** | | | | | | | | | | | | **Sexual risk behaviors** | | | | | | | | | | | | |  |
| --- | --- | --- | --- | --- | --- | --- | --- | --- | --- | --- | --- | --- | --- | --- | --- | --- | --- | --- | --- | --- | --- | --- | --- | --- | --- | --- |
|  | **Study year** | | | **Study location** | **Study base*** | **Sample method#** | | | **Male(%)** | **Age range (Mean)** | **Marital status¶** | | **Time span** | | **Premarital sex** | | | **Commercial sex** | | | | **Multiple sex partners** | | | |  |
|  |  |  |  |  |  |  |  |  |  |  |  |  |  |  | **n** | **N** | **%** | **n** | **N** | | **%** | **n** | **N** | | **%** | |
| Liu, 1998 [[114](#_ENREF_114)] | | 1997 | Anhui | | CR | | PBS | 50 | | 15-45 | M:83% | Lifetime | | 202 | | 886 | 22.8 | − | | − | − | 68 | | 886 | 7.7 | |
|  | | 1997 | Yunnan | | CR | | PBS | − | | 15-69 | M:59% | Last month | | − | | − | − | − | | − | 0.8 | − | | − | − | |
|  | | 1998 | Yunnan | | CR | | PBS | − | | 15-69 | M:59% | Last month | | − | | − | − | − | | − | 2.5 | − | | − | − | |
| Xia, 2004 [[115](#_ENREF_115)] | | 1999 | Hainan | | CR | | PBS | 0 | | 18-49 (34) | M:95% | Lifetime | | 89 | | 606 | 14.7 | 0 | | 606 | 0.0 | − | | − | − | |
|  | | 1999 | Yunnan | | CR | | PBS | − | | 15-69 | M:59% | Last month | | − | | − | − | − | | − | 1.2 | − | | − | − | |
| Parish, 2003 [[116](#_ENREF_116)] | | 2000 | Nationwide | | CR | | PBS | − | | 20-64 | − | Last year | | − | | − | − | − | | − | − | 211 | | 2373 | 8.9 | |
| Wang, 2002 [[117](#_ENREF_117)] | | 2000 | Shanghai | | OY | | PBS | 61 | | 17-24 | NM:100% | Lifetime | | 239 | | 1304 | 18.3 | − | | − | − | − | | − | − | |
| Yu, 2003 [[118](#_ENREF_118)] | | 2001 | Gansu | | CR | | PBS | 74 | | 20-49 | − | Lifetime | | − | | − | − | − | | − | − | 275 | | 850 | 32.4 | |
| Zhang, 2004 [[119](#_ENREF_119)] | | 2001 | Jilin | | OY | | PBS | 50 | | 15-24 | NM:100% | Lifetime | | 172 | | 1083 | 15.9 | − | | − | − | − | | − | − | |
| Liu, 2005 [[120](#_ENREF_120)] | 2003 | | | Anhui | OY | CS | | | 50 | 17-37 (24) | NM:100% | Lifetime | | | 350 | 605 | 57.9 | − | − | | − | 59 | | 605 | 9.8 | |
| Tang, 2009 [[121](#_ENREF_121)] | 2003 | | | Sichuan | CR | PBS | | | 0 | 20-49 (34) | M:100% | Lifetime | | | − | − | − | − | − | | − | 153 | | 2000 | 7.7 | |
| Yang, 2007 [[122](#_ENREF_122)] | 2003 | | | Yunnan | CR | PBS | | | 45 | 18-55 | M:88% | Lifetime | | | − | − | − | 138 | 4759 | | 2.9 | − | | − | − | |
| Chang, 2007 [[123](#_ENREF_123)] | 2005 | | | Liaoning | OY | PBS | | | 54 | 15-24 (21) | NM:92% | Lifetime | | | 248 | 584 | 42.5 | 70 | 248 | | 28.2 | − | | − | − | |
| Fu, 2011 [[124](#_ENREF_124)] | 2005 | | | Yunnan | CR | PBS | | | 53 | 16-55 (35) | M:79% | Last 4 years | | | − | − | − | 8 | 591 | | 1.4 | 179 | | 591 | 30.3 | |
| Ji, 2007 [[125](#_ENREF_125)] | 2005 | | | Anhui | CR | PBS | | | 33 | 25-55 | M:93% | | Last year | | − | − | − | 30 | 1997 | | 1.5 | − | | − | − | |
| Niu, 2007 [[126](#_ENREF_126)] | 2005 | | | Henan | CR | PBS | | | 48 | 18-60 (34) | M:85% | | Last year | | 94 | 852 | 11.0 | − | − | | − | 41 | | 784 | 5.2 | |
| Zhao, 2006 [[127](#_ENREF_127)] | 2005 | | | Nationwide | CR | PBS | | | 63 | 15-64 | M:0% | | Lifetime | | 113 | 709 | 15.9 | − | − | | − | − | | − | − | |
| Pan, 2011[[128](#_ENREF_128)] | 2006 | | | Nationwide | CR | PBS | | | − | 15-49 | − | | Lifetime | | 719 | 2066 | 34.8 | 95 | 1377 | | 6.9 | − | | − | − | |
| Hong, 2009 [[129](#_ENREF_129)] | 2007 | | | Anhui | CR | PBS | | | 0 | 18-49 (31) | M:100% | | Lifetime | | − | − | − | − | − | | − | 5 | | 737 | 0.7 | |
| Qin, 2009 [[130](#_ENREF_130)] | 2007 | | | Anhui | CR | PBS | | | 0 | 18-49 (36) | M:100% | | Last year | | − | − | − | − | − | | − | 87 | | 1873 | 4.6 | |
| Xiong, 2010 [[131](#_ENREF_131)] | 2007 | | | Hubei | OY | PBS | | | 45 | 23-40 | M:80% | | Lifetime | | 8 | 56 | 14.3 | − | − | | − | − | − | | − | |

**Table S1: Studies reporting sexual risk behaviors among population groups that do not belong to the classical high risk groups in China** *(continued)***.**

| **First author, publication year** | **Study design** | | | | | | | **Sexual risk behaviors** | | | | | | | | | | | | |  |
| --- | --- | --- | --- | --- | --- | --- | --- | --- | --- | --- | --- | --- | --- | --- | --- | --- | --- | --- | --- | --- | --- |
|  | **Study year** | **Study location** | **Study base*** | **Sample method#** | **Male(%)** | **Age range (Mean)** | **Marital status¶** | **Time span** | **Premarital sex** | | | **Commercial sex** | | | | **Multiple sex partners** | | | | |  |
|  |  |  |  |  |  |  |  |  | **n** | **N** | **%** | **n** | **N** | **%** | | **n** | **N** | | | **%** | |
| Li, 2010 [[132](#_ENREF_132)] | 2008 | Shanxi | CR | PBS | 40 | 18-60 (40) | M:84% | Lifetime | − | − | − | − | − | | − | 109 | 1660 | | 6.6 | |  |
| Li, 2010 [[133](#_ENREF_133)] | 2008 | Yunnan | CR | CS | 100 | (58) | M:78% | Last month | − | − | − | 22 | 165 | | 13.3 | − | − | − | | |  |
| Ma, 2010 [[134](#_ENREF_134)] | 2008 | Gansu | OY | PBS | 64 | 15-24 (20) | M/C:33% | Last year | 123 | 230 | 53.5 | − | − | | − | 46 | 123 | 37.4 | | |  |
| Xuan, 2010 [[135](#_ENREF_135)] | 2008 | Henan | OY | PBS | − | 14-28 | NM:39% | Lifetime | 37 | 86 | 43.0 | − | − | | − | 27 | 222 | 12.2 | | |  |
| He, 2011 [[136](#_ENREF_136)] | 2009 | 4 provinces | CR | PBS | 45 | 20-35 | NM:100% | Lifetime | 411 | 1631 | 25.2 | − | − | | − | − | − | − | | |  |
| Pan, 2011 [[137](#_ENREF_137)] | 2009 | Sichuan | OY | PBS | 65 | 14-28 (21) | − | Lifetime | 157 | 604 | 26.0 | 20 | 604 | | 3.3 | 26 | 310 | 8.4 | | |  |
| Zhang, 2011 [[138](#_ENREF_138)] | 2009 | Shanghai | OY | PBS | 50 | 15-24 (19) | NM:100% | Lifetime | 765 | 6023 | 12.7 | − | − | | − | − | − | − | | |  |

* RWs: routine workers from venues: e.g. factories, restaurants, retail shops and markets; TD: truck drivers; CR: community residents; IP: in transit individuals; MR: migrant workers returned to rural areas; U: university/universities; OY: out-of-school youth; PC: participants recruited from clinical settings, i.e. gynecological or obstetric clinics and premarital medical check-up centers.

# CS: convenience sampling; QS: quota sampling; PBS: probability-based sampling (i.e. cluster sampling, stratified sampling, multi-stage sampling).

**¶** M: married; NM: never married; C: cohabiting.

**Table S2: Studies reporting condom usage among population groups that do not belong to the classical high risk groups in China.** Each section represents different study populations.

| **First author, publication year** | **Study design** | | | | | | **Condom usage** | | | | | | | |
| --- | --- | --- | --- | --- | --- | --- | --- | --- | --- | --- | --- | --- | --- | --- |
|  | **Study year** | **Study location** | **Study base*** | **Sampling method#** | **Male (%)** | **Age range (Mean)** | **Marital status¶** | **Type of sexual partners** | **Time span** | **n** | **N** | | **%** | |
|  |  |  |  |  |  |  |  |  |  |  |  |  |  |  |
| ***Floating population*** | |  |  |  |  |  |  |  |  |  | |  | |  |
| Chen, 2006 [[139](#_ENREF_139)] | 2000 | Anhui | TD | CS | 100 | 15-51 (28) | M: 72% | Unspecified | Last time | 149 | 418 | | 35.6 | |
| NIMH, 2007 [[4](#_ENREF_4)] | 2001 | Fujian | RW | PBS | 48 | 18-40 | M/C: 78% | Unspecified | Last 3 months | 129 | 1204 | | 10.7 | |
| Li, 2004 [[6](#_ENREF_6)] | 2002 | Beijing | RW | QS | 66 | 18-30 (25) | NM: 48% | Unspecified | Last three times | 211 | 992 | | 21.3 | |
|  | 2002 | Jiangsu | RW | QS | 66 | 18-30 (26) | NM: 41% | Unspecified | Last three times | 289 | 1161 | | 24.9 | |
| Liu, 2004 [[140](#_ENREF_140)] | 2002 | Guangdong | RW | QS | 0 | 16-25 (19) | M: 74% | Unspecified | Last time | 7 | 192 | | 3.6 | |
| Sun, 2004 [[8](#_ENREF_8)] | 2002 | Sichuan | TD | PBS | − | − | M: 90% | Commercial | Last year | 82 | 266 | | 30.8 | |
|  |  |  |  |  |  |  |  | Casual | Last time | 53 | 164 | | 32.3 | |
| Lin, 2006 [[9](#_ENREF_9)] | 2003 | Beijing | RW | QS | − | 18-30 | NM: 77% | Unspecified | Last three times | 212 | 900 | | 23.6 | |
| Zheng, 2006 [[141](#_ENREF_141)] | 2004 | Beijing | RW | CS | 100 | 18-40 (28) | M: 65% | Unspecified | Last year | 30 | 648 | | 4.6 | |
| Zheng, 2006 [[142](#_ENREF_142)] | 2004 | Shanxi | CR | CS | 89 | 18-50 (30) | − | Unspecified | Last time | 131 | 394 | | 33.2 | |
|  |  |  |  |  |  |  |  |  | Last year | 76 | 394 | | 19.3 | |
| Ding, 2006 [[143](#_ENREF_143)] | 2005 | Chongqing | CR | PBS | 53 | 13-58 (29) | NM: 51% | Casual | Last year | 11 | 78 | | 14.1 | |
| Xiao, 2007 [[12](#_ENREF_12)] | 2005 | Guangdong | RW | CS | 47 | 20-26: 50% | NM: 63% | Casual | Last year | 29 | 129 | | 22.5 | |
| Zhang, 2007 [[13](#_ENREF_13)] | 2005 | Guangdong | RW | PBS | 0 | > 15 (24) | NM: 100% | Unspecified | Last 6 months | 98 | 233 | | 42.1 | |
| Zhao, 2005 [[14](#_ENREF_14)] | 2005 | Yunnan | RW | CS | 100 | 15-51 (28.4) | M: 70% | Commercial | Last time | 5 | 21 | | 23.8 | |
| Gao, 2010 [[15](#_ENREF_15)] | 2006 | Yunnan | RW | PBS | 100 | 17-64 (32) | M: 63% | Commercial | Last time | 77 | 116 | | 66.4 | |
|  |  |  |  |  |  |  |  |  | Last year | 59 | 116 | | 50.9 | |
| Li, 2007 [[16](#_ENREF_16)] | 2006 | Guangdong | TD | PBS | 100 | 21-62 (36) | M/C: 82% | Commercial | Last year | 94 | 153 | | 61.4 | |
| Li, 2008 [[144](#_ENREF_144)] | 2006 | Tianjin | RW | PBS | 66 | 16-65 (32) | M: 67% | Unspecified | Last year | 261 | 551 | | 47.4 | |
| Li, 2008 [[19](#_ENREF_19)] | 2006 | Mongolia | RW | PBS | 41 | 16-65 (33) | M/C: 73% | Commercial | Last year | 16 | 44 | | 36.4 | |

**Table S2: Studies reporting condom usage among population groups that do not belong to the classical high risk groups in China** *(continued)*.

| **First author, publication year** | **Study design** | | | | | | **Condom usage** | | | | | | | |
| --- | --- | --- | --- | --- | --- | --- | --- | --- | --- | --- | --- | --- | --- | --- |
|  | **Study year** | **Study location** | **Study base*** | **Sampling method#** | **Male (%)** | **Age range (Mean)** | **Marital status¶** | **Type of sexual partners** | **Time span** | **n** | | **N** | | **%** |
|  |  |  |  |  |  |  |  |  |  |  |  |  |  |  |
| Li, 2010 [[20](#_ENREF_20)] | 2006 | Guangdong | CR | PBS | 41 | 15-49 | M: 55% | Unspecified | Last year | 426 | | 812 | | 52.5 |
| He, 2009 [[22](#_ENREF_22)] | 2007 | Shanghai | RW | QS | 44 | 18-34: 76% | M: 61% | Unspecified | Last month | 334 | | 1756 | | 19.0 |
| Mantell, 2011 [[25](#_ENREF_25)] | 2007 | Jiangsu | RW | CS | 44 | 18-57 (23) | M: 22% | Casual | Last month | 70 | | 182 | | 38.5 |
|  |  |  |  |  |  |  |  | Unspecified | Last time | 209 | | 370 | | 56.5 |
| Zhang, 2008 [[145](#_ENREF_145)] | 2007 | Shandong | MR | PBS | 54 | 15-69 (36) | − | Casual | Last 3 months | 36 | | 128 | | 28.1 |
| Chen, 2010 [[26](#_ENREF_26)] | 2008 | Sichuan | CR | CS | 43 | 20-40: 53% | M: 73% | Commercial | Last time | − | | − | | 60.0 |
|  |  |  |  |  |  |  |  | Casual | Last time | − | | − | | 31.3 |
| Liang, 2009 [[146](#_ENREF_146)] | 2008 | Guangdong | RW | QS | 39 | < 35: 91% | − | Casual | Last year | 113 | | 249 | | 45.4 |
| Lin, 2010 [[30](#_ENREF_30)] | 2008 | Guangdong | RW | PBS | 39 | 20-30: 60% | NM: 40% | Casual | Last 6 months | 5 | | 12 | | 41.7 |
| Meng, 2010 [[147](#_ENREF_147)] | 2008 | Jilin | RW | PBS | 90 | 18-48 (34) | M: 70% | Casual | Last time | 36 | | 325 | | 11.1 |
|  |  |  |  |  |  |  |  | Casual | Last year | 21 | | 325 | | 6.5 |
| Ren, 2011 [[31](#_ENREF_31)] | 2008 | Shanghai | RW | PBS | 100 | 16-71 (39) | M: 81% | Unspecified | Last 3 months | 115 | | 980 | | 11.7 |
|  |  |  |  |  |  |  |  | Casual | Last 3 months | 16 | | 30 | | 53.3 |
| Tan, 2011 [[32](#_ENREF_32)] | 2008 | Guangxi | RW | PBS | 89 | 18-48 (32) | M: 51% | Unspecified | Last 6 months | 130 | | 785 | | 16.6 |
| Wei, 2010 [[148](#_ENREF_148)] | 2008 | Henan | RW | CS | − | 17-40: 85% | M: 54% | Unspecified | Last time | 59 | | 380 | | 15.5 |
| Zhang, 2010 [[33](#_ENREF_33)] | 2008 | Sichuan | IP | CS | 85 | 17-66 (35) | M/C: 86% | Commercial | Last time | 32 | | 81 | | 39.5 |
|  |  |  |  |  |  |  |  | Commercial | Last year | 22 | | 81 | | 27.2 |
|  |  |  |  |  |  |  |  | Casual | Last time | 24 | | 66 | | 36.4 |
|  |  |  |  |  |  |  |  | Casual | Last year | 15 | | 66 | | 22.7 |
| Du, 2011 [[149](#_ENREF_149)] | 2009 | Chongqing | RW | PBS | 85 | 20-50: 86% | M/C: 87% | Commercial | Last time | 20 | | 35 | | 57.1 |
|  |  |  |  |  |  |  |  | Commercial | Last year | 13 | 35 | | 37.1 | |
|  |  |  |  |  |  |  |  | Casual | Last time | 4 | 19 | | 21.1 | |
|  |  |  |  |  |  |  |  | Casual | Last year | 2 | 19 | | 10.5 | |

**Table S2: Studies reporting condom usage among population groups that do not belong to the classical high risk groups in China** *(continued)*.

| **First author, publication year** | **Study design** | | | | | | **Condom usage** | | | | | | | | | |  |  |  |
| --- | --- | --- | --- | --- | --- | --- | --- | --- | --- | --- | --- | --- | --- | --- | --- | --- | --- | --- | --- |
|  | **Study year** | **Study location** | **Study base*** | **Sampling method#** | **Male (%)** | **Age range (Mean)** | **Marital status¶** | **Type of sexual partners** | **Time span** | **n** | | **N** | | | **%** | |  |  |  |
| Li, 2011 [[34](#_ENREF_34)] | 2009 | Anhui | RW | PBS | 60 | 20-50: 79% | M: 58% | Commercial | Last time | 53 | 83 | | | 63.9 | | |  |  |  |
|  |  |  |  |  |  |  |  | Commercial | Last year | 24 | 86 | | | 27.9 | | |  |  |  |
| S un, 2011 [[150](#_ENREF_150)] | 2009 | 2 provinces | RW | PBS | 56 | 15-24 (21) | M: 21% | Unspecified | Last time | 211 | 333 | | | 63.4 | | |  |  |  |
| Wang, 2011 [[151](#_ENREF_151)] | 2009 | Shanghai | RW | PBS | 96 | 16-65 (40) | M/C: 84% | Casual | Last 3 months | 12 | | 17 | | | 70.6 | |  |  |  |
| Wei, 2010 [[35](#_ENREF_35)] | 2009 | Chongqing | RW | PBS | 73 | 18-46 (35) | M/C: 84% | Commercial | Last time | 112 | | | 185 | | | 60.5 | | | |
|  |  |  |  |  |  |  |  | Commercial | Last year | 62 | | | 185 | | | 33.5 | | | |
|  |  |  |  |  |  |  |  | Casual | Last time | 57 | | | 98 | | | 58.2 | | | |
|  |  |  |  |  |  |  |  | Casual | Last year | 34 | | | 98 | | | 34.7 | | | |
| Zhang, 2010 [[36](#_ENREF_36)] | 2009 | Guangdong | RW | PBS | 51 | 18-30 | M: 33% | Casual | Last year | 11 | | | 83 | | | 13.3 | | | |
|  |  |  |  |  |  |  |  |  | Last time | 52 | | | 83 | | | 62.7 | | | |
| ***College students*** | |  |  |  |  |  |  |  |  |  | | |  | | |  | |  |  |
| Cottrell, 2004 [[46](#_ENREF_46)] | 2001 | Jiangsu | 19 U | PBS | − | − | − | Unspecified | Last time | 57 | | | 133 | | | 42.9 | | |  |
| Ma, 2006 [[54](#_ENREF_54)] | 2003 | Zhejiang | 2 U | PBS | 50 | − | − | Unspecified | Last time | 1217 | | | 2524 | | | 48.2 | | |  |
|  |  |  |  |  |  |  |  |  | Last year | 764 | | | 2015 | | | 37.9 | | |  |
| Chen, 2005 [[55](#_ENREF_55)] | 2004 | Fujian | 8 U | PBS | 43 | − | − | Unspecified | Last time | 169 | | | 302 | | | 56.0 | | |  |
| Cai, 2006 [[60](#_ENREF_60)] | 2005 | Anhui | 1 U | PBS | 57 | − | − | Unspecified | Last month | 71 | | | 147 | | | 48.3 | | |  |
| Li, 2007 [[65](#_ENREF_65)] | 2005 | Sichuan | 1 U | PBS | 51 | 17-35 (20) | − | Unspecified | Last time | − | | | − | | | 12.3 | | |  |
| Tan, 2007 [[68](#_ENREF_68)] | 2005 | Hubei | 1U | PBS | 49 | − | − | Unspecified | Last time | − | | | − | | | 35.7 | | |  |
| Xu, 2007 [[71](#_ENREF_71)] | 2005 | Guangxi | 4 U | PBS | 45 | 15-29 (21) | − | Unspecified | Last time | 45 | | | 143 | | | 31.5 | | |  |
| Wu, 2007 [[77](#_ENREF_77)] | 2006 | Zhejiang | 1 U | PBS | 69 | M:22 | − | Commercial | Last year | 12 | | | 37 | | | 32.4 | | |  |
|  |  |  |  |  |  |  |  | Unspecified | Last time | 215 | | | 407 | | | 52.8 | | |  |
| Zhou, 2010 [[89](#_ENREF_89)] | 2007 | Jiangxi | 3 U | PBS | 51 | 17-24 (20) | − | Unspecified | Last time | 58 | | | 151 | | | 38.4 | | |  |
|  |  |  |  |  |  |  |  |  | Last 3 months | 39 | | | 151 | | | 25.8 | | |  |

**Table S2: Studies reporting condom usage among population groups that do not belong to the classical high risk groups in China** *(continued)*.

| **First author, publication year** | **Study design** | | | | | | **Condom usage** | | | | | | |
| --- | --- | --- | --- | --- | --- | --- | --- | --- | --- | --- | --- | --- | --- |
|  | **Study year** | **Study location** | **Study base*** | **Sampling method#** | **Male (%)** | **Age range (Mean)** | **Marital status¶** | **Type of sexual partners** | **Time span** | **n** | **N** | **%** | |
| Wang, 2011 [[108](#_ENREF_108)] | 2009 | Chongqing | 1 U | PBS | 100 | 17-24 (20.6) | C: 0.5% | Unspecified | Last time | 45 | 82 | 54.9 | |
|  |  |  |  |  |  |  |  |  | Last month | 30 | 68 | 44.1 | |
| ***Other groups*** | |  |  |  |  |  |  |  |  |  |  |  |  |
| Sun, 2001 [[113](#_ENREF_113)] | 1996 | Jiangsu | CR | PBS | − | 18-30 (24) | − | Unspecified | Last time | 23 | 270 | 8.5 | |
| Wang, 2000 [[152](#_ENREF_152)] | 1998 | Sichuan | CR | PBS | 100 | 16-35 | − | Casual | Last year | 38 | 99 | 38.4 | |
| Chen, 2006 [[153](#_ENREF_153)] | 2002 | Fujian | PC | CS | 0 | M:27 | M:100% | Unspecified | Last time | 33 | 362 | 9.1 | |
| Tang, 2009 [[121](#_ENREF_121)] | 2003 | Sichuan | CR | PBS | 0 | 20-49 (34) | M:100% | Unspecified | Last 3 months | 118 | 2000 | 5.9 | |
| Zhang, 2007 [[154](#_ENREF_154)] | 2004 | Hebei | CR | PBS | 0 | 20-52 (37) | M:100% | Unspecified | Last year | 222 | 2178 | 10.2 | |
| Fu, 2011 [[124](#_ENREF_124)] | 2005 | Yunnan | CR | PBS | 53 | 16-55 (34.6) | M:79% | Casual | Last 4 years | 20 | 90 | 22.2 | |
| Ji, 2007 [[125](#_ENREF_125)] | 2005 | Anhui | CR | PBS | 33 | 25-55 | M:93% | Commercial | Last year | 35 | 149 | 23.5 | |
| Niu, 2007 [[126](#_ENREF_126)] | 2005 | Henan | CR | PBS | 48 | 18-60 (34) | M:85% | Unspecified | Last year | 37 | 784 | 4.7 | |
| Xu, 2007 [[155](#_ENREF_155)] | 2006 | Anhui | CR | PBS | 51 | 15-49 (33) | − | Casual | Last time | 20 | 30 | 66.7 | |
| Ye, 2008 [[156](#_ENREF_156)] | 2007 | Anhui | CR | PBS | 0 | 18-49 (35.6) | M:100% | Unspecified | Last year | 13 | 382 | 3.4 | |
| Li, 2010 [[133](#_ENREF_133)] | 2008 | Yunnan | CR | CS | 100 | M:58 | M:78% | Commercial | Last month | 5 | 22 | 22.7 | |
|  |  |  |  |  |  |  |  | Unspecified | Last month | 13 | 165 | 7.9 | |
| Ma, 2010 [[134](#_ENREF_134)] | 2008 | Gansu | OY | PBS | 64 | 15-24 (20) | M/C:33% | Commercial | Last time | 32 | 46 | 69.6 | |
| Zhou, 2010 [[157](#_ENREF_157)] | 2008 | Shanghai | CR | PBS | 58 | 18-60 (39) | M:81% | Unspecified | Last time | 354 | 1744 | 20.3 | |
|  |  |  |  |  |  |  |  |  |  |  |  |  | |

* RWs: routine workers from venues: e.g. factories, restaurants, retail shops and markets; TD: truck drivers; CR: community residents; IP: in transit individuals; MR: migrant workers returned to rural areas; U: university/universities; OY: out-of-school youth; PC: participants recruited from clinical settings, i.e. gynecological or obstetric clinics and premarital medical check-up centers.

# CS: convenience sampling; QS: quota sampling; PBS: probability-based sampling (i.e. cluster sampling, stratified sampling, multi-stage sampling).

**¶** M: married; NM: never married; C: cohabiting.

**Table S3: Studies reporting STIs among population groups that do not belong to the classical high risk groups in China.** Each section represents different study populations

| **First author, publication year** | **Study design** | | | | | | | **STDs** | | | | | | | | | | | |
| --- | --- | --- | --- | --- | --- | --- | --- | --- | --- | --- | --- | --- | --- | --- | --- | --- | --- | --- | --- |
|  | **Study year** | **Study location** | **Study base*** | **Sampling method#** | **Male (%)** | **Age range (Mean)** | **Marital status¶** | **Chlamydia** | | | | **Syphilis** | | | | **Gonorrhea** | | | |
|  |  |  |  |  |  |  |  | **TM§** | **n** | **N** | **%** | **TM§** | **n** | **N** | **%** | **TM§** | **n** | **N** | **%** |
| ***Floating population*** | |  |  |  |  |  |  |  |  |  |  |  |  |  |  |  |  |  |  |
| Detels, 2003 [[2](#_ENREF_2)] | 2001 | Fujian | RW | PBS | 48 | 18-40 | M/C: 78% | LCR | 124 | 1316 | 9.4 | RPR,  TPPA | 24 | 1316 | 1.8 | LCR | 14 | 1316 | 1.1 |
| NIMH, 2007 [[4](#_ENREF_4)] | 2001 | Fujian | RW | PBS | 48 | 18-40 | M/C: 78% | PCR | 124 | 1478 | 8.4 | TPPA | 25 | 1520 | 1.6 | PCR | 13 | 1485 | 0.9 |
| He, 2005 [[158](#_ENREF_158)] | 2002 | Shanghai | RW | CS | 100 | 16-66 (33.6) | M: 92% | LCR | 34 | 972 | 3.5 | TPHA | 8 | 841 | 0.9 | LCR | 5 | 972 | 0.5 |
| Hu, 2004 [[5](#_ENREF_5)] | 2002 | Anhui | RW | PBS | 49 | 25-44: 75% | M: 84% | PCR | 12 | 333 | 3.6 | RPR | 1 | 333 | 0.3 | − | − | − | − |
| Ye, 2003 [[159](#_ENREF_159)] | 2002 | Shanghai | RW | − | 100 | 20-35 | − | LCR | 38 | 1128 | 3.4 | TRUST, TPPA | 10 | 1041 | 1.0 | LCR | 8 | 1128 | 0.7 |
| Hesketh, 2005 [[160](#_ENREF_160)] | 2004 | Zhejiang | RW | PBS | 51 | 14-67 (24) | M/C: 52% | − | − | − | − | VDRL, TPPA | 20 | 4148 | 0.5 | − | − | − | − |
| Ge, 2007 [[161](#_ENREF_161)] | 2005 | Shanghai | RW | PBS | 81 | 17-63 (36) | − | − | − | − | − | RPR, TPHA | 8 | 571 | 1.4 | − | − | − | − |
| Zhao, 2005 [[14](#_ENREF_14)] | 2005 | Yunnan | RW | CS | 100 | 15-51 (28.4) | M: 70% | PCR | 17 | 182 | 9.3 | − | − | − | − | PCR | 1 | 182 | 0.6 |
| Li, 2007 [[16](#_ENREF_16)] | 2006 | Guangdong | TD | PBS | 100 | 21-62 (36) | M/C: 82% | − | − | − | − | TRUST | 2 | 257 | 0.8 | − | − | − | − |
| ***College students*** | |  |  |  |  |  |  |  |  |  |  |  |  |  |  |  |  |  |  |
| Zhang, 2007 [[73](#_ENREF_73)] | 2005 | Sichuan | 2 U | PBS | 0 | 16-28 (21.2) | − | − | − | − | − | − | − | − | − | − | 7 | 1615 | 0.4 |
| Wu, 2007 [[77](#_ENREF_77)] | 2006 | Zhejiang | 1 U | PBS | 69 | (22) | − | − | − | − | − | RPR | 0 | 1608 | 0.0 | − | − | − | − |
| ***Other groups*** | |  |  |  |  |  |  |  |  |  |  |  |  |  |  |  |  |  |  |
| Hodgson, 1988 [[162](#_ENREF_162)] | 1986 | Zhejiang | PC | CS | 0 | 18-55 | NM: 7% | DFA | 10 | 1000 | 1.0 | − | − | − | − | − | − | − | − |
| Ni, 1990 [[163](#_ENREF_163)] | 1989 | Beijing | PC | CS | 0 | − | − | DFA | 43 | 607 | 7.1 | − | − | − | − | − | − | − | − |

**Table S3: Studies reporting STIs among population groups that do not belong to the classical high risk groups in China** *(continued)***.**

| **First author, publication year** | **Study design** | | | | | | | **STDs** | | | | | | | | | | | |
| --- | --- | --- | --- | --- | --- | --- | --- | --- | --- | --- | --- | --- | --- | --- | --- | --- | --- | --- | --- |
|  | **Study year** | **Study location** | **Study base*** | **Sampling method#** | **Male (%)** | **Age range (Mean)** | **Marital status¶** | **Chlamydia** | | | | **Syphilis** | | | | **Gonorrhea** | | | |
|  |  |  |  |  |  |  |  | **TM§** | **n** | **N** | **%** | **TM§** | **n** | **N** | **%** | **TM§** | **n** | **N** | **%** |
| Yang, 1990 [[164](#_ENREF_164)] | 1989 | Liaoning | PC | − | 0 | − | − | − | − | − | − | − | − | − | − | LCR | 7 | 500 | 1.4 |
| Jiang, 1994 [[165](#_ENREF_165)] | 1992 | Liaoning | CR | − | 39 | 19-59 | − | DFA | 12 | 249 | 4.8 | − | − | − | − | − | − | − | − |
| Li, 1997 [[166](#_ENREF_166)] | 1994 | Yunnan | CR | PBS | 0% | − | M: 100% | DFA | 33 | 664 | 5.0 | − | − | − | − | − | − | − | − |
| Wu, 2000 [[167](#_ENREF_167)] | 1997 | Fujian | PC | PBS | 50 | − | − | − | − | − | − | TRUST, TPPA | − | − | 1.2 | − | − | − | − |
|  | 1998 | Fujian | PC | PBS | 50 | − | − | − | − | − | − | TRUST, TPPA | − | − | 1.7 | − | − | − | − |
|  | 1999 | Fujian | PC | PBS | 50 | − | − | − | − | − | − | TRUST, TPPA | 59 | 2872 | 2.1 | − | − | − | − |
| Deng, 2000 [[168](#_ENREF_168)] | 1998 | Guangdong | PC | PBS | 0 | − | − | − | − | − | − | VDRL, TPHA | 1 | 4400 | 0.0 | − | 1 | 4400 | 0.0 |
|  | 1999 | Guangdong | PC | PBS | 0 | − | − | − | − | − | − | VDRL, TPHA | 4 | 4545 | 0.1 | − | − | − | − |
| Parish, 2003 [[116](#_ENREF_116)] | 2000 | Nationwide | CR | PBS | 52 | 20-64 | − | LCR | 78 | 2373 | 3.3 | − | − | − | − | − | − | − | − |
| Hesketh, 2005 [[169](#_ENREF_169)] | 2001 | Zhejiang | PC | PBS | 50 | − | − | − | − | − | − | TPHA | 49 | 8910 | 0.6 | − | − | − | − |
|  | 2001 | Shanxi | PC | PBS | 50% | − | − | − | − | − | − | TPHA | 16 | 4562 | 0.4 | − | − | − | − |
|  | 2001 | Yunnan | PC | PBS | 50% | − | − | − | − | − | − | TPHA | 24 | 3742 | 0.6 | − | − | − | − |
| Yu, 2003 [[118](#_ENREF_118)] | 2001 | Gansu | CR | PBS | 74% | 20-49 | − | − | − | − | − | TRUST | 30 | 850 | 3.5 | − | − | − | − |
| Chen, 2006 [[153](#_ENREF_153)] | 2002 | Fujian | PC | CS | 0 | (27) | M: 100% | PCR | 51 | 504 | 10.1 | TPPA | 1 | 504 | 0.2 | PCR | 4 | 504 | 0.8 |

**Table S3: Studies reporting STIs among population groups that do not belong to the classical high risk groups in China** *(continued)***.**

| **First author, publication year** | **Study design** | | | | | | | **STDs** | | | | | | | | | | | |
| --- | --- | --- | --- | --- | --- | --- | --- | --- | --- | --- | --- | --- | --- | --- | --- | --- | --- | --- | --- |
|  | **Study year** | **Study location** | **Study base*** | **Sampling method#** | **Male (%)** | **Age range (Mean)** | **Marital status¶** | **Chlamydia** | | | | **Syphilis** | | | | **Gonorrhea** | | | |
|  |  |  |  |  |  |  |  | **TM§** | **n** | **N** | **%** | **TM§** | **n** | **N** | **%** | **TM§** | **n** | **N** | **%** |
| Cheng, 2007 [[170](#_ENREF_170)] | 2002 | Shenzhen | PC | PBS | 0 | − | − | − | − | − | − | TRUST, TPPA | 189 | 58785 | 0.3 | − | − | − | − |
|  | 2003 | Shenzhen | PC | PBS | 0 | − | − | − | − | − | − | TRUST, TPPA | 555 | 118235 | 0.4 |  | − | − | − |
|  | 2004 | Shenzhen | PC | PBS | 0 | − | − | − | − | − | − | TRUST, TPPA | 637 | 141619 | 0.5 | − | − | − | − |
|  | 2005 | Shenzhen | PC | PBS | 0 | − | − | − | − | − | − | TRUST, TPPA | 827 | 159017 | 0.5 | − | − | − | − |
| Wen, 2003 [[171](#_ENREF_171)] | 2002 | Shanghai | CR | PBS | 0 | 36-49: 80% | M: 100% | DFA | 109 | 1631 | 6.68 | − | − | − | − | − | 2 | 1631 | 0.12 |
| He, 2007 [[172](#_ENREF_172)] | 2003 | Hubei | PC | PBS | 0 | (27) | M: 100% | PCR | 102 | 519 | 19.7 | − | − | − | − | PCR | 33 | 519 | 6.4 |
|  | 2004 | Hubei | PC | PBS | 0 | − | − |  | 64 | 422 | 15.2 | − | − | − | − | − | 36 | 422 | 8.5 |
|  | 2005 | Hubei | PC | PBS | 0 | − | − |  | 43 | 377 | 11.4 | − | − | − | − | − | 17 | 377 | 4.5 |
| Tang, 2009 [[121](#_ENREF_121)] | 2003 | Sichuan | CR | PBS | 0% | 20-49 (34) | M: 100% | LCR | 127 | 2000 | 6.4 | RPR, TPHA | 9 | 2000 | 0.5 | GC | 33 | 2000 | 1.7 |
| Hesketh, 2005 [[169](#_ENREF_169)] | 2004 | Zhejiang | CR | CS | 47 | 16-71 (33) | M/C: 80% | − | − | − | − | VDRL, TPHA | 15 | 2197 | 0.7 | − | − | − | − |
| Franceschi, 2007 [[173](#_ENREF_173)] | 2005 | Shanxi | CR | PBS | 0% | 20-44 | − | PCR | 8 | 399 | 2.0 | − | − | − | − | PCR | 0 | 399 | 0.0 |
| Dai, 2012 [[174](#_ENREF_174)] | 2009 | Sichuan | CR | PBS | 45% | 15-49: 73% | M/C: 83% | − | − | − | − | RPR, TPHA | 2 | 4950 | 0.0 | − | − | − | − |

* RWs: routine workers from venues: e.g. factories, restaurants, retail shops and markets; TD: truck drivers; CR: community residents; IP: in transit individuals; MR: migrant workers returned to rural areas; U: university/universities; OY: out-of-school youth; PC: participants recruited from clinical settings, i.e. gynecological or obstetric clinics and premarital medical check-up centers.

# CS: convenience sampling; QS: quota sampling; PBS: probability-based sampling (i.e. cluster sampling, stratified sampling, multi-stage sampling).

¶ M: married; NM: never married; C: cohabiting.

§TM: testing methods. Chlamydia infection was tested based on nucleic acid hybridization by Ligase Chain Reaction (LCR), or nucleic acid amplification by Polymerase Chain Reaction (PCR), or Direct Fluorescent Antibody test (DFA); syphilis infection was tested based on Toluidine Red Unheated Serum Test (TRUST), or Treponema Pallidum Particle Agglutination assay (TPPA), or Venereal Disease Research Laboratory test (VDRL), or Rapid Plasma Reagin test (RPR), or Treponema Pallidum Hemagglutination Assay (TPHA); gonorrhea infection was tested based on similar methods as Chlamydia, i.e. LCR, PCR, or Gonorrhea Culture (GC).

Reference

1. Anderson AF, Qingsi Z, Hua X, Jianfeng B (2003) China's floating population and the potential for HIV transmission: a social-behavioural perspective. AIDS Care 15: 177-185.

2. Detels R, Wu Z, Rotheram MJ, Li L, Guan J, et al. (2003) Sexually transmitted disease prevalence and characteristics of market vendors in eastern China. Sex Transm Dis 30: 803-808.

3. Li L, Wu Z, Rotheram-Borus MJ, Guan J, Yin Y, et al. (2009) Visiting entertainment venues and sexual health in China. Arch Sex Behav 38: 814-820.

4. Group NCHSPT (2007) Sexually transmitted disease and HIV prevalence and risk factors in concentrated and generalized HIV epidemic settings. AIDS 21 Suppl 2: S81-90.

5. Hu Z, Wang DB, Cao HT, Zhang XJ (2004) STDs/AIDS knowledge, attitude and practice among market vendors of congregating trading market in Hefei. Chin J Public Health 20: 1479-1480.

6. Li X, Fang X, Lin D, Mao R, Wang J, et al. (2004) HIV/STD risk behaviors and perceptions among rural-to-urban migrants in China. AIDS Educ Prev 16: 538-556.

7. Lou CH, Shen Y, Gao ES, Tu XW (2005) Sexual behaviors of unmarried migrants with different characteristics. Reproduction & Contraception 25.

8. Sun Q, Zhang JX, Li XS, Lan YJ, Che XG, et al. (2004) [Study on AIDS related risk behaviors and the correlated factors among three groups of population in Sichuan province]. Zhonghua Liu Xing Bing Xue Za Zhi 25: 761-765.

9. Lin XY, Fang XY, Lin DH, Li XM (2006) An analysis of HIV/STD high risk sexual behaviors and HIV/STD knowledge in mobile population in Beijing. Chin J AIDS STD 12: 426-428.

10. Xie LC, Zhong YL, Zeng XC (2006) Investigation on status of reproductive health among floating married women aged childbearing in Shenzhen. China Journal of Family Planning 7: 417-420.

11. Zhao QG, Li B, Tian FL, Yuan DM, Chen CL, et al. (2006) Study on the current status and relative factors on reproductive health of unmarried floating population in Huizhou city. Chin J Epidemiol 27: 1038-1042.

12. Xiao ZJ, Cai WD, Tan JG (2007) A cross-sectional study on KAP about AIDS/HIV among the workers in Shenzhen from other places. Modern Preventive Medicine 34: 2441-2446.

13. Zhang JD, Lian W, Jia GZ, Guo YP (2007) Investigation of the sexual health situation of unmarried non-resident young women in Guangzhou city. Journal of Sexual Health 32: 4581-4583.

14. Zhao R, Gao H, Shi X, Tucker JD, Yang Z, et al. (2005) Sexually transmitted disease/HIV and heterosexual risk among miners in townships of Yunnan Province, China. AIDS Patient Care STDS 19: 848-852.

15. Gao YF (2010) HIV/AIDS risk analysis among migrant workers at a highway construction site in western Yunnan. Population Research 34: 96-106.

16. Li H, Chen QH, Feng XH, Xie JY, Zhang SP (2007) Investigation on AIDS related KAP among truck drivers in Shenzhen. Chin J Public Health 23: 725-726.

17. Li X, Zhang L, Stanton B, Fang X, Xiong Q, et al. (2007) HIV/AIDS-related sexual risk behaviors among rural residents in China: potential role of rural-to-urban migration. AIDS Educ Prev 19: 396-407.

18. Liu LF, Jiang SA, Zheng YY, Hu PC, Chen FZ (2007) A survey on the population sociological features and AIDS related KAB among rural migrants workers. Modern Preventive Medicine 34: 2048-2050.

19. Li B, Wang Y, Zhang F, Gao B, Wei LQ (2008) Study on extramarrital sexual intercourse and condom use related factors among floating population. Chin J Public Health 24: 791-792.

20. Li YY, Li N, Zhou Y, Liu BY, Wu JQ (2010) Study of HIV/AIDS-related sexual behaviors and influencing factors among migrants in Shenzhen, China. Fudan Univ J Med Sci 37: 304-309.

21. Xin QQ, Huang Y, Hu CX, Wang JF, Huang F (2010) Investigation of HIV/AIDS related behaviors and condom use among construction workers. Chin J Public Health 26: 914.

22. He N, Zhang J, Yao J, Tian X, Zhao G, et al. (2009) Knowledge, attitudes, and practices of voluntary HIV counseling and testing among rural migrants in Shanghai, China. AIDS Educ Prev 21: 570-581.

23. He N, Cao H, Yin Y, Gao M, Zhang T, et al. (2009) Herpes simplex virus-2 infection in male rural migrants in Shanghai, China. Int J STD AIDS 20: 112-114.

24. Li SF, Zhong CH, Lei X, Zhang M, Xu XL (2009) A study on knowledge, attitude and behavior of AIDS/STD among migrant workers in Chongqing. Chongqing Medicine 38: 2002-2004.

25. Mantell JE, Kelvin EA, Sun X, Zhou J, Exner TM, et al. (2011) HIV/STI risk by migrant status among workers in an urban high-end entertainment centre in Eastern China. Health Educ Res 26: 283-295.

26. Chen W, Li Q, Liu L, Dong LM, Luan RS (2010) AIDS-related KAP and the related factors among migrant people in some middle-scaled tourism city. Modern Preventive Medicine 37: 502-504.

27. Gao SH, Y XM, Gong LX, Wang B, Yu M, et al. (2009) Gender difference in sexual and reproductive health among young migrant workers. Chin J Public Health 25: 913-915.

28. Huang X, Xu L, Zhang X, Calzavara L (2011) A study on effects of living environment on migrant construction workers' cognition to AIDS in former Nanhui district of Shanghai. J Environ Occup Med 28: 785-788.

29. Li N, Wang XL, Li XM (2012) Sexual behavior and its influencing factors among farmer workers. Chin J Public Health 28: 166-168.

30. Lin AH, Wang BB, Shen SY, Ling L (2010) The current situation of the knowledge, attitude and behavior concerning AIDS of the migrant workers in Guangzhou. Modern Preventive Medicine 37: 1281-1283.

31. Ren JM, Calazvara L, Fang H, Kang LY (2011) Situation and influencing factors of sexual behaviors among male construction workers in Shanghai, China. Chin J Public Health 27: 347-348.

32. Tan SK, Qiu XQ, Li SL, Zhao D (2011) Study on knowledge, attitude, behavior and its influencing factors of AIDS among migrant workers in the areas with a high incidence of HIV. Modern Preventive Medicine 38: 900-902.

33. Zhang Q, Huang Y, Lv F, Jiang XS (2010) Analysis on behavior related to HIV of migrant workers in Nanchong Modern Preventive Medicine 37: 363-366.

34. Li XJ, Zhu YB, Feng JB, Sun J, Yao H (2011) Knowledge and prevalence of AIDS/STD among floating population in Hefei city. Chin J Public Health 27: 1572-1574.

35. Wei SL, Zhou SJ, Qin ZY, Li D, Wang QY (2010) Survey on HIV/AIDS related prevention knowledge, attitudes and high-risk behavior among migrant workers in Chongqing. China Journal of Family Planning 7: 411-413.

36. Zhang Y, Yao HY, Sun JF, Feng Q, Wang Q (2010) A cross-sectional study on HIV/AIDS related knowledge, attitude and behavior among new generation of migrant workers in Shenzhen. China Journal of Family Planning 11: 667-669.

37. Zhang YH, Gan K, Meng YC, Cheng YM (2011) Analysis on sexual behaviors of unmarried female migrants. Modern Preventive Medicine 38: 2079-2081.

38. Zuo LD, Wu WX, Zhao RQ, Cai JL, Xu L (2011) Survey on sexual behavior and attitude among male floating population in Guangzhou. China Journal of Family Planning 19: 409-412.

39. He JL, Wang HY, Cui G, Zhang B (1997) Study on sexual issues among university student. Chin J AIDS STD 3: 103-105.

40. Wu JQ, Qiu LX, Zhao PF, Gao ES (1997) Comparison of sexual education and sex-related KAP between medical students in Shanxi and Shanghai, China. Chin J Public Health 13: 632-634.

41. Li AL, Li LM, Zhang YC, Wang AZ (1999) A survey on STDs/AIDS knowledge, perception and sexual behavior among university students in Beijing. Chin J Public Health 15: 545-546.

42. Tao FB, Zhang HB, Xu SJ, Zeng GY (1999) An epidemiological study on health risk behavior of college students. Chin J Sch Health 20: 249-250.

43. Wang SM, Huang JH, Wang LK (2000) A survey on HIV/AIDS related issues among college students in Shanghai. Chin J Sch Health 21: 172-173.

44. Xia SJ (2000) A study on sexual attitude and behaviors among college students in Guangzhou. Chin J Prev Med 34: 315.

45. Zhang LY, Gao X, Dong ZW, Tan YP, Wu ZL (2001) Premarital sexual activities among students in a university in Beijing, China. Sex Transm Dis 29: 212.

46. Cottrell L, Li X, Stanton B, Harris C, D'Alessandri D, et al. (2004) Perceptions regarding preventive sexual practices and communication with sexual partners among Chinese college students. Preventive Medicine 40: 189-196.

47. Xiang WN, Liu WD, Lu Y (2003) A survey on HIV/AIDS related KAP among college students in Guangzhou. Chin J School Health 24: 165-166.

48. Yang XB, Wang DB, Hong Q, et al (2010) Analyzing the results of AIDS/STD behavioral surveillance in four provinces of health project The Chinese Health Service Management 9: 631-634.

49. Fan CX, Ma SB, Wu CP, Chen J, Wang HS, et al. (2004) A study on sexual related KAP among college students. Chin J Public Health 20: 275-276.

50. Huang J, Carol B, Kristopher PF, Angela R, Ann BW (2005) Knowledge, Attitudes, Behaviors, and Perceptions of Risk Related to HIV/AIDS among Chinese University Students in Hunan, China AIDS Patient Care and STDs 19: 769-777.

51. Song YM, Xu G, Huang F, Zhu ZP, Ye DQ (2003) Investigation on knowledge, attitude and behavior about AIDS among medical university students. Chin J Public Health 19: 374-376.

52. Lin PX, Huang BT, Li SB, Chen QS (2009) Survey on AIDS knowledge, attitude, behavior and practices among university students and evaluation of educational effect. Modern Preventive Medicine 36: 3495-3497.

53. Dong LL, Li XB, Li XY, Liu MQ, Liu YL, et al. (2005) A cross sectional study of sexual knowledge, attitude, behavior among colledge students. Reproductive health 23: 3113-3115.

54. Ma Q, Ono-Kihara M, Cong L, Xu G, Zamani S, et al. (2006) Sexual behavior and awareness of Chinese university students in transition with implied risk of sexually transmitted diseases and HIV infection: a cross-sectional study. BMC Public Health 6: 232.

55. Chen G, Lin X, Yang Y, Yan PP, Zheng J, et al. (2005) A survey of AIDS-related knowledge, attitude and behavior among college students in 4 cities in Fujian, China. Strait J Prev Med 11: 19-21.

56. Guan Z, Bai CY, Zhang D, Chen R (2006) KAP of HIV/AIDS among college students in Shenyang. Chin J Sch Health 27: 123-125.

57. Lonn E, Sahlholm K, Maimaiti R, Abdukarim K, Andersson R (2007) A traditional society in change encounters HIV/AIDS: knowledge, attitudes, and risk behavior among students in northwestern China. AIDS Patient Care STDS 21: 48-56.

58. Sun L, Zhu HB, Zhang CY, Li JK, Zhao P, et al. (2006) The current situation of health risk behaviors among adolescents in Sichuan province, China. Chin J Sch Health 27: 1069-1072.

59. Zhai L, Sun BJ, Wang XZ, Zhang L, Shen YF (2007) A study of unhealthy behaviors among 1138 college students in Beijing. Chin J Sch Health 28: 264-266.

60. Cai HY, Wang DB, Cheng J, Huang ZL, Guan JF, et al. (2006) Correlation study on attachment styles and HIV/AIDS prevention behaviors among medical students. Chin J Sch Health 27: 748-750.

61. Chen B, Lu YN, Wang HX, Ma QL, Chen P (2008) Sexual and reproductive health service needs of university/college students: updates from a survey in Shanghai, China. Asian J Androl 10: 607-615.

62. Kong XM ZC, Zhang JJ, Guo Q (2007) KAP of premarital sexual behaviors among 146 undergraduates in Beijing. Chin J Sch Health 28: 788-789.

63. Li J, Jiang LJ, Wang WY, Jiang Y (2007) The gender differences of AIDS-related knowledge, attitude and practice among college students Modern Preventive Medicine 34: 1529-1531.

64. Li SY, Xu DL, Lu ZX (2007) Investigation on sexuality and contraceptive practice of the minority nationality female university students. China Journal of Maternal and Child Health Care 23: 3300-3302.

65. Li T, Li L, Mao XY, Gong Y, Yu H (2007) Investigation on AIDS related KAP among college students. Modern Preventive Medicine 34: 2407-2409.

66. Sun SM, Wang BL, Yu DH, Liu XF (2007) KAP on AIDS among university students and senior high school students in Tongzhou district in Beijing. Chin J Sch Health 28: 683-684.

67. Song Y, Ji CY (2010) Sexual intercourse and high-risk sexual behaviours among a national sample of urban adolescents in China. J Public Health (Oxf) 32: 312-321.

68. Tan XD , Pan JJ, Zhou D, Wang CH, Xie CJ (2007) HIV/AIDS Knowledge, Attitudes and Behaviors Assessment of Chinese Students: A Questionnaire Study. Int J Environ Res Public Health 4: 248-253.

69. Xu XY, Mao ZF, Duan GF, Li SY (2007) Investigation on sexual behavior among 985 female medical students in Wuhan, China. Chinese Journal of Mental Health 21: 752.

70. Xu QY, Geng JG, Li L, Liu XH, Zhu JH, et al. (2007) Survey on KAP about sex among college freshmen Modern Preventive Medicine 34: 1532-1533.

71. Xu YF, Zhou J, Mo XJ, Zhu JJ, Li P (2007) STD/AIDS-related knowledge, attitude and behavior among college students in Guangxi. Chin J Sch Health 28: 974-975.

72. Zeng LN LB, Qin LJ (2007) A survey on HIV/AIDS related KAP among students in two colleges in Guangzhou. Chin J Sch Health 28: 646-647.

73. Zhang Y (2007) Procreation health of 3196 unmarried female university students. Chin J Sch Health 28: 109-110.

74. Zhao LY (2006) A survey of HIV/AIDS related KAP among college students in Handan. Modern Preventive Medicine 33: 1427.

75. Zhou W, Zhang H, Meng XP (2006) Survey on AIDS knowledge, attitude and behavior among students of two universities in Beijing. Chin J AIDS STD 12: 143-150.

76. Liu YH, Sun FF (2007) Investigation on HIV/AIDS related KAP among college students. Chin J Public Health 23: 1064.

77. Wu JM, Pan XH, Yang JW, Xu Y (2007) Research on the risk sexual behavior and HIV infection in students. Chin Pre Med 10: 604-606.

78. Wang K, Wang YF, Zhang ZY, Ma RL (2007) A survey of sexual KAP among students in China University of Geosciences. Chin J Sch Health 28: 1025-1026.

79. Sun XY, Shi YH, Wang PY, Chang C (2010) AIDS related knowledge, attitude, and behavior and influencing factors among college students in China. Chin J Sch Health 31: 270-273.

80. Wang L, Ding ZW, Yan RX (2010) HIV/AIDS epidemic situation and data analysis among young students from 2006-2009 in China. Chin J Epidemiol 31: 1017-1021.

81. Xu DL LZ (2011) Investigation on sexual behaviors among female college students majoring in liberal arts and history in Wuhan. Chin J Public Health 27: 518.

82. Yan H, Chen W, Wu H, Bi Y, Zhang M, et al. (2009) Multiple sex partner behavior in female undergraduate students in China: a multi-campus survey. BMC Public Health 9: 305.

83. Liu XG, Jin GY, Lv J, Hong SH (2009) Cognition and attitude about antemarital sexual behavior and abortion of college students from a college in Hangzhou. Chin J Sch Health 30: 804-806.

84. Peng BH (2009) Characteristics of contemporary college students' sexual behavior and its relationship with sexual attitudes and knowledge. Journal of Capital University of Economics 4: 100-105.

85. Wang C, Ling L, He Q (2009) Survey on the knowledge, attitude and practice of HIV/AIDS among university students in Guangzhou city, Guangdong Province. Chinese Journal of Health Education 25: 119-121.

86. Cheng Y, Lou CH, Gao ES (2010) Influence of exposure to sexual information in the mass media on college students' sexual opinions and behavior in Shanghai. Reproduction & Contraception 30: 543-547.

87. Duan AX, Zhao FX, Liu RH, Liu H, Mu YQ (2011) Survey on the knowledge, attitudes and highly risky behaviors of AIDS among university students and needs assessment of health education. Modern Preventive Medicine 38: 1050-1054.

88. Zhou YZ, Xiong CL, Ying P (2007) Survey of status and requirement about sexual behavior and contraception among unmarried college/university students in China. Acta Med Univ Sci Technol Huazhong 38: 561-571.

89. Zhou XJ, Xi QH, Ruan SY (2010) Evaluation of AIDS comprehensive intervention effect for students in private college. Modern Preventive Medicine 37: 4079-4081.

90. Zhou Y, Xiong J, Li J, Huang S, Shang X, et al. (2011) Urgent need for contraceptive education and services in Chinese unmarried undergraduates: a multi-campus survey. J Huazhong Univ Sci Technolog Med Sci 31: 426-432.

91. Chen F, Xu YP (2009) Sex knowledge, attitude and sexual behaviors in college students Chin J Public Health 25: 1029-1030.

92. Pan LP, Wang QF (2009) Survey on sexual knowledge and behaviors among medical and nonmedical college students Chin J Public Health 25: 1037.

93. Albrektsson M, Alm L, Tan XD, Andersson R (2009) HIV/AIDS awareness, attitudes and risk behavior among university students in Wuhan, China. Open AIDS J 3: 55–62.

94. Wu CY, Xie CZ (2009) Health risk behavior among adolescent in urban areas of Zaozhuang city. Chin J Sch Health 30: 807-809.

95. Zhang D, Yang YJ, Zhang X, Chen F, Guo L, et al. (2009) A survey on unhealthy behaviors among college students in Shenyang. Chin J Prev Med 17: 419-420.

96. Zhang M, Wang YX, Li SF, Yang JL, Zhong CH (2010) Survey on sexual health knowledge, sexual behavior and attitude of some universities in Chongqing. Chongqing Medicine 39: 565-568.

97. You Q ZY, Du M (2010) Investigation on sexual perception and behavior of medical college students and sexual education. Journal of Harbin Medical University 44: 165-167.

98. You Q ZY, Du M (2010) Investigation on sexual behavior and contraception among unmarried college students. Journal of Harbin Medical University 44: 409-412.

99. Wu XT (2009) Survey on AIDS knowledge, attitude and behavior among students of two colleges in Xinyang. Modern Preventive Medicine 36: 2916-2922.

100. Zhu HB, Li JY, Wang YD, Tian YY, Du YY, et al. (2009) A cross sectional study on unhealthy behaviors among adolescents in Zhangye Chin J Sch Health 30: 1133-1135.

101. Yang X, Xiong LN, Liu ZX, Wang J, Yuan L, et al. (2010) Survey on contraceptive method among college students in Nanchang. Chinese Journal of Maternal and Child Health Care 4: 519-521.

102. Yang L, Li X, Jin T, Xiong ZB (2010) Study on the current status of sexual behaviors among the college students in Chengdu. China Journal of Family Planning 1: 36-38.

103. Chen YT, Luo XM, QIn W, Liu AD, Tian H, et al. (2010) Survey on AIDS related behaviors and knowledge among medical students. Chin J Public Health 26: 1447-1448.

104. Shi XD, Lu J, Liang HN, Lv S (2010) Epidemiologic studies on health hazards behaviors of adolescents in Nanning. Modern Preventive Medicine 37: 1297-1303.

105. Chen YT QW, Luo XM (2011) Investigation of college students AIDS and sex related behaviors, knowledge and attitudes in Zunyi. Modern Preventive Medicine 38: 661-663.

106. Liu YM SY, Liu N, Hu WB, Zhao Y (2011) Survey on sexual knowledge, attitude and behaviors among college students in Suzhou. Chin J Sch Health 23: 91-93.

107. Wang ZY, Huang LC (2010) Survey on knowledge, attitude and behavior about AIDS/STD among nonmedical college students. Acta Med Univ Sci Technol Huazhong 39: 577-581.

108. Wang L ML, Liang H (2011) Awareness situation of AIDS prevention knowledge and reproductive health status of medical male students. Chinese General Practice 14: 1126-1129.

109. Yang WQ, Zhang HC (2011) Research on influencing factors to premarital sexual behavior of female students. Chinese Journal of Maternal and Child Health Care 26: 2298-2300.

110. Zhu AL GL, Gu XL, Shang JH, Yuan WH (2011) Investigation on risk factors of premarial sex and sex abuse among female college students Chinese Journal of Maternal and Child Health Care 26: 1066-1067.

111. Guo J, Zhang S (2011) Investigation about knowledge, attitude and behavior of reproductive health among college students in Beijing. China Journal of Family Planning 19: 89-93.

112. Ma J, Chen L, Shu G (2001) [Dynamic surveillance of risk behaviors facilitating sexually transmitted disease/acquired immunodeficiency syndrome transmission among permanent residents in Kunming city in 1996-1999]. Zhonghua Liu Xing Bing Xue Za Zhi 22: 323-325.

113. Sun XM HC, Wen Y, Tao B, (2001) A community-based AIDS education intervention study in Kunshan country. Chin J Epidemiol 22: 326-328.

114. Liu H, Xie J, Yu W, Song W, Gao Z, et al. (1998) A study of sexual behavior among rural residents of China. J Acquir Immune Defic Syndr Hum Retrovirol 19: 80-88.

115. Xia DY LS, He QY (2004) A questionnaire-based survey on attitude and behavior of sex among rural women in Hainan province. Chin J Epidemiol 25: 586-589.

116. Parish WL, Laumann EO, Cohen MS, Pan S, Zheng H, et al. (2003) Population-based study of chlamydial infection in China: a hidden epidemic. JAMA 289: 1265-1273.

117. Wang B LC, Shen Y, Gao ES, Xu XW (2002) Sexual behavior and contraceptive use among unmarried youths in sub-urban Shanghai. Reproduction & Contraception 22: 99-106.

118. Yu AL WJ, Gou WB (2003) Survey on HIV/STD related KABP among residents in the Tibet autonomous prefecture of south Gansu province. Chin J Public Health 19: 1355.

119. Zhang LY, Jejeebhoy S, Shah IH, Zhang LH, Hsia J, et al. (2004) Access to contraceptive services among unmarried young people in the north-east of China. Eur J Contracept Reprod Health Care 9: 147-154.

120. Liu H, Detels R, Li X, Stanton B, Hu Z, et al. (2005) Risk of HIV transmission within marriage in rural China: implications for HIV prevention at the family level. Sex Transm Dis 32: 418-424.

121. Tang YJ, Samuelson J, Qingsheng D, Ali MM, Li X, et al. (2009) The prevalence of sexually transmitted and other lower reproductive tract infections among rural women in Sichuan Province, China. Southeast Asian J Trop Med Public Health 40: 1038-1047.

122. Yang X, Derlega VJ, Luo H (2007) Migration, behaviour change and HIV/STD risks in China. AIDS Care 19: 282-288.

123. Chang C CL, Sun TW (2007) Holistic view on vulnerability of HIV/AIDS infection among youth out of schools. Journal of Peking University (Health Sciences) 39: 132-135.

124. Fu Z, He N, Duan S, Jiang Q, Ye R, et al. (2011) HIV infection, sexual behaviors, sexual networks, and drug use among rural residents in Yunnan Province, China. AIDS Behav 15: 1017-1025.

125. Ji G, Detels R, Wu Z, Yin Y (2007) Risk of sexual HIV transmission in a rural area of China. Int J STD AIDS 18: 380-383.

126. Niu GY ZY, Chen ZW (2007) Knowledge, attitude and behaviour analysis on AIDS among 852 workers of food, public and drinking water. Modern Preventive Medicine 34: 105-107.

127. Zhao FM, Wang LH, Guo SF (2006) Investigation on sexual behavior with non-spouse and condom use among reproductive age men and women. Chin J Public Health 22: 1309-1310.

128. Pan S, Parish WL, Huang Y (2011) Clients of female sex workers: a population-based survey of China. J Infect Dis 204 Suppl 5: S1211-1217.

129. Hong H, Qin QR, Li LH, Ji GP, Ye DQ (2009) Condom use among married women at risk for sexually transmitted infections and HIV in rural China. Int J Gynaecol Obstet 106: 262-265.

130. Qin QR, Ji GP, Xu J, Jiang QC, Hong H, et al. (2009) Risk of sexual HIV transmission among wives left behind and wives of nonmigrant men in rural areas of China. J Assoc Nurses AIDS Care 20: 308-315.

131. Xiong JW YP, Guan Huang (2010) Analysis on contraception status and service requirements of high-income youth in Wuhan. China Journal of Maternal and Child Health Care 25: 785-789.

132. Li XM MC, Lv AL, Guo XY (2010) The study of AIDS-related knowledge attitude and behaviors in resource-limited rural residents of Shanxi province. Chin J Nurs 45: 389-393.

133. Li CW PY, Long XD, Li H, Duo L (2010) Investigation on the knowledge and behavior among a male AIDS patients above 50 years old in Kaiyuang and Yingjiang. Modern Preventive Medicine 37: 1073-1075.

134. Ma JX ZF, Li YQ, Liu ZQ (2010) Investigation on HIV/AIDS risk factors among youth out of school in Baiyin. Chin J Sch Health 31: 479-480.

135. Xuan ZD CW (2010) Investigation on sexual behaviors and condom use among youths out of schools Chinese Health Service Management 2: 125-127.

136. He H, Cao G (2011) Status and influencing factors of premarital sexual behaviour among youth in the cities of China. Chin J Fam Plann 19: 665-668.

137. Pan CM TX, Song Z, Zhang XF (2011) Investigation on AIDS related knowledge, attitudes and risk behaviors among the adolescents outside school. Modern Preventive Medicine 38: 4908-4913.

138. Zhang P LC (2011) Analysis on factors influencing adolescent sex-related behaviors in Shanghai city under the structural equation model. China Health Statistics 28: 139-141.

139. Chen XS, Yin YP, Gong XD, Liang GJ, Zhang WY, et al. (2006) Prevalence of sexually transmitted infections among long-distance truck drivers in Tongling, China. Int J STD AIDS 17: 304-308.

140. Liu SF, Xu RH, Wang SY (2004) Evaluation on effects of HIV/AIDS education among mobile female factory workers. Chin J Public Health 20: 945-946.

141. Zheng ZZ, Zhou Y (2006) Condom uses and its determinants of male migrants in urban China. China Journal of Family Planning 12: 730-732.

142. Zheng QQ, Li WX, Gao HY, Sha RN, Jiang YN (2006) Analysis of a comdom promotion trial among floating population. Chin J Public Health 22: 515.

143. Ding XB, Chen H, Pan CB, Zeng Y, Wang YL (2006) Analysis on knowledge, attitude and features of risk behavior about AIDS among floating population. Chin J Public Health 22: 1293-1294.

144. Li XJ, Gu Q, Yuan YY, Wang G (2008) Study on the impact factors of condom usage among the floating population China J Epidemiol

29: 1171.

145. Zhang YQ, Shu HY, Liu DY, Kong Y, Li XT (2008) Investigation on AIDS prevention, infection situation and behavior in peasant workers. Chin J Public Health 24: 8-9.

146. Liang BL, Cai JS, Feng HF, Liang HM, Liu FG (2009) STD-related knowledge and non-marriage sexual behavior among urban peasant workers. Chin J Public Health 25: 1147-1149.

147. Meng XJ, Wang L, Meng XD, Ding GW, Guo W, et al. (2010) Investigation on AIDS prevention, infection situation and behavior among peasant workers. Modern Preventive Medicine 37: 2696-2699.

148. Wei JT, Zhu W (2010) Evaluating the effect of AIDS health education on migrant workers. The Chinese Health Service Management 4: 279-287.

149. Du JX, Xiong HY (2011) Analysis on the effect of health education and intervention about AIDS among construction migrant workers in a district of Chongqing. Chongqing Medicine 40: 2859-2864.

150. Sun XY, Shi YH, Ji W (2011) Survey on reproductive health among young migrants in Tianjin and Shanxi provinces. Chin J Fam Plann 19: 22-25.

151. Wang WH, Cao Z, Xu Y, Calzavara L (2011) Evaluation of the effects on KAP for AIDS prevention among construction workers in Huangpu district after intervention with different health education measures. J Environ Occup Med 28: 730-734.

152. Wang SM, Gao MY (2000) Employment and contextual impact of safe and unsafe sexual practices for STI and HIV: the situation in China. Int J STD AIDS 11: 536-544.

153. Chen XS, Yin YP, Chen LP, Thuy NT, Zhang GY, et al. (2006) Sexually transmitted infections among pregnant women attending an antenatal clinic in Fuzhou, China. Sex Transm Dis 33: 296-301.

154. Zhang HJ YW, Dong YP, Liu MY (2007) Study on usage and knowledge on condom among women at childbearing age in rural area. China Journal of Maternal and Child Health Care 22: 2108-2109.

155. Xu L, Fang JG, Wang HD (2007) Investigation on knowledge and behavior of country town residents in Anhui province. Chin J Public Health 23: 1437-1438.

156. Ye R, Ren NJ, Jin W (2008) Status of condom use and related factors among married women in rural area. Chin J Public Health 24: 20-21.

157. Zhou XL, Lu CY, Chen WQ (2010) Knowledge, attitude and behavior related to AIDS/STD among rural residents in Guangzhou. Chin J Public Health 26: 1087-1088.

158. He N, Detels R, Zhu J, Jiang Q, Chen Z, et al. (2005) Characteristics and sexually transmitted diseases of male rural migrants in a metropolitan area of Eastern China. Sex Transm Dis 32: 286-292.

159. Ye W, Fang J, Tian ZG, Zhang XH (2003) Analysis of the sexually transmitted disease infection among migrant workers of a certain district in Shanghai. Shanghai J of Med Lab Sci 18: 372-373.

160. Hesketh TM, Lu L (2005) HIV and syphilis in Chinese internal migrants. AIDS 19: 1550.

161. Ge XQ, Cao XN, Zhang HL, Chen X (2007) Investigation on the characteristics of STD/AIDS infections among floating construction workers. Chinese Journal of Health Education 23: 741-743.

162. Hodgson JE, Shi YF, Gao YL, Wu KJ, Jiang BY, et al. (1988) Chlamydial infection in a Chinese gynecologic outpatient clinic. Obstet Gynecol 71: 96-100.

163. Ni AP, Gu CX, Li ST, Yang HY, Wang B, et al. (1990) The prevalence of Chlamydia infection in outpatient clinics in Beijing, China. Genitourin Med 66: 125.

164. Yang JL JF, Chen SR, Zhang YK, Liu Y (1990) Survey on STDs among gynecological clinic outpatients. Chin J Public Health 4: 173.

165. Jiang RH, Zhang ZG, Zhao JW (1994) Investigation on the present status of infections with Chlamydia trachomatis, Mycoplasma hominis and Ureaplasma urealyticum in patients with venereal diseases, sexual abusers and healthy people in Dalian. Chinese Journal of Epidemiology 15: 87-89.

166. Li W, Wang TY, He CY (1997) The study on the epidemiological features and risk factor of Chamydia Trachomatis reproductive tract infection among 664 married women at reproductive age in rural areas. Chinese Journal of Epidemiology 18: 77-79.

167. Wu HM (2000) Survey on the prevalence of syphilis among 5300 premarital couples for medical check in Nanping. Chin J Public Health 16: 1022.

168. Deng QP (2000) Investigation on sexually transmitted diseases among 8754 pregnant women. Journal of Practical Nursing 16: 23.

169. Hesketh T, Tang F, Wang ZB, Huang XM, Williams D, et al. (2005) HIV and syphilis in young Chinese adults: implications for spread. Int J STD AIDS 16: 262-266.

170. Cheng JQ, Zhou H, Hong FC, Zhang D, Zhang YJ, et al. (2007) Syphilis screening and intervention in 500,000 pregnant women in Shenzhen, the People's Republic of China. Sex Transm Infect 83: 347-350.

171. Wen ZY, Wu JQ, Zhong XH (2003) Study on relationship between reproductive tract infections and personal hygiene behaviors among vulnerable married women in Shanghai. Reproduction & Contraception 23: 340-344.

172. He AJ ZL, Chen XG (2007) Investigation on sexually transmitted diseases among females. China Journal of Maternal and Child Health Care 22: 2827-2828.

173. Franceschi S, Smith JS, van den Brule A, Herrero R, Arslan A, et al. (2007) Cervical infection with Chlamydia trachomatis and Neisseria gonorrhoeae in women from ten areas in four continents. A cross-sectional study. Sex Transm Dis 34: 563-569.

174. Dai S, Shen Z, Zha Z, Leng R, Qin W, et al. (2012) Seroprevalence of HIV, syphilis, and hepatitis C virus in the general population of the Liangshan Prefecture, Sichuan Province, China. J Med Virol 84: 1-5.
