# Supplementary material for: Trends in High-Risk Sexual Behaviors among General Population Groups in China: A Systematic Review
Source: PLoS One. 2013 Nov 13;8(11):e79320. doi: 10.1371/journal.pone.0079320 (PMC3827370; doi:10.1371/journal.pone.0079320)
Supplement: File S4 — Results of generalized linear mixed model analysis (GLMM). (DOCX) [file pone.0079320.s004.docx]

**Supplementary materials**

**File S4: Results of generalized linear mixed model analysis (GLMM)**

We relate the proportion/prevalence (*P_i_*) of each indicator of high-risk sexual behavior to year of study by the function as follows: logit (*P_i_*) = α+ β * (Year of study-2000) + γ*_i_*, where γ*_i_* ~N (0, σ^2^). γ*_i_* is a random effect term, which takes into account heterogeneity of study designs of the various studies. Dashed lines in the Fig. 2-4 reflect average proportion/prevalence trends, which only consider α and β. Parameters of α, β, and σ^2^ are shown in the following table:

| Indicator of high-risk sexual behavior | |  | α | (95% CI) | β | (95% CI) | p-value | σ^2^ |
| --- | --- | --- | --- | --- | --- | --- | --- | --- |
|  | *Floating population* | | | | | | | |
| Premarital sex | | Fig. 2a | -0.94 | (-1.75, -0.14) | 0.00 | (-0.12, 0.13) | 0.94 | 0.31 |
| Commercial sex | | Fig. 2b | -2.75 | (-3.52, -1.98) | 0.07 | (-0.04, 0.19) | 0.20 | 0.43 |
| Multiple sex partners | | Fig. 2c | -2.48 | (-3.21, -1.75) | 0.12 | (-0.03, 0.26) | 0.10 | 0.92 |
| Condom use at the last intercourse | | Fig. 2d | -1.11 | (-3.28, 1.07) | 0.11 | (-0.16, 0.39) | 0.39 | 0.75 |
| Condom use during the last month to year | | Fig. 2d | -0.89 | (-3.92, 2.14) | -0.16  -0.16 | (-0.56, 0.24) | 0.40 | 2.24 |
|  | *College students* | | | | | | | |
| Premarital sex | | Fig. 3a | -2.22 | (-2.49, -1.96) | 0.03 | (-0.02, 0.07) | 0.19 | 0.39 |
| Multiple sex partners | | Fig. 3b | -0.90 | (-3.68, 1.89) | -0.16 | (-0.61, 0.28) | 0.44 | 1.87 |
| Condom use at the last intercourse | | Fig. 3c | -0.24 | (-1.13, 0.65) | 0.02 | (-0.14, 0.18) | 0.77 | 0.14 |
|  | *Other, more general groups* | | | | | | | |
| Premarital sex among out-of-school youth | | Fig. 4a | -1.01 | (-2.44, 0.42) | 0.02 | (-0.20, 0.25) | 0.82 | 0.84 |
| Commercial sex among community residents | | Fig. 4b | -6.50 | (-10.64, -2.36) | 0.59 | (-0.29, 1.46) | 0.12 | 0.96 |
| Multiple sex partners among community residents | | Fig. 4b | -2.28 | (-3.32, -1.24) | -0.07  -0.07 | (-0.28, 0.14) | 0.45 | 1.33 |
| Chlamydia among women recruited from gynlogical clinics | | Fig. 4c | -2.57 | (-3.10, -2.04) | 0.07 | (-0.01, 0.15) | 0.069 | 0.55 |
